# The intracellular region of truncated neurotrophin receptor TrkB-T1 is sufficient to promote brain glial reactivity, neuroinflammation and neurotoxicity

**DOI:** 10.1186/s43556-026-00650-x

**Published:** 2026-11-13

**Authors:** Lola Ugalde-Triviño, María C. Serrano, Margarita Díaz-Guerra

**Affiliations:** 1 Instituto de Investigaciones Biomédicas Sols-Morreale (IIBM), Consejo Superior de Investigaciones Científicas-Universidad Autónoma de Madrid, 28029 Madrid, Spain; 2 Instituto de Ciencia de Materiales de Madrid (ICMM), Consejo Superior de Investigaciones Científicas, 28049 Madrid, Spain

**Keywords:** Cell-penetrating peptides, Excitotoxicity, Intranasal administration, Neuroinflammation, Stroke, TrkB-T1

## Abstract

The development of advanced therapies for stroke, spinal cord injury, and neurodegenerative diseases—primary causes of death, disability and dementia—requires a comprehensive understanding of the interplay between excitotoxicity, aberrant neurotrophic-signalling, glial reactivity, and neuroinflammation. Recent evidence has identified the truncated neurotrophin receptor TrkB-T1 as a potential master regulator of these processes. TrkB-T1 levels increase after stroke whereas the pro-survival full-length isoform (TrkB-FL) decreases. Furthermore, stroke-induced excitotoxicity triggers TrkB-T1 regulated intramembrane proteolysis (RIP), which produces TrkB-T1-ICD, a poorly characterised fragment containing the receptor intracellular domain and a few transmembrane amino acids. This study investigated whether the TrkB-T1 intracellular domain intrinsically contributes to neurotoxicity, reactive gliosis, and neuroinflammation. Our results predict that TrkB-T1 RIP generates a nuclear localization signal in TrkB-T1-ICD that might support nuclear translocation and injury-induced transcriptional regulation in neurons undergoing excitotoxicity. Using cell-penetrating peptides (CPPs), we generated a TrkB-T1-ICD mimetic peptide (Bio-LTT1_Ct_) containing the TrkB-T1 intracellular region. Bio-LTT1_Ct_ translocated to the nucleus of neurons and astrocytes, provoking cell death and inducing early transcriptional changes in neurons, resembling those triggered by excitotoxicity. In vivo, intranasally administrated Bio-LTT1_Ct_ was brain-permeable and distributed within cortical neurons and astrocytes across both hemispheres. Notably, Bio-LTT1_Ct_ administration was sufficient to promote TrkB isoform imbalance, glial reactivity, and acquisition of proinflammatory profiles, all considered stroke hallmarks. In conclusion, these results imply that TrkB-T1 RIP might be a central mechanism of ischaemic damage and identify the receptor’s intracellular region as sufficient to recapitulate stroke-like effects, marking this pathological mechanism as a critical target for therapy.

**Supplementary Information:**

The online version contains supplementary material available at https://doi.org/10.1186/s43556-026-00650-x.

## Introduction

Stroke is the second leading cause of death worldwide and a major cause of dementia and disability [1]. Ischaemic stroke (≈ 85% of cases) results from cerebral blood vessel occlusion, causing severe hypoperfusion, nutrient/oxygen deprivation, and irreversible neuronal death in the infarct core. The surrounding penumbra experiences less severe hypoperfusion and remains metabolically active but functionally impaired. This tissue can persist viable for several hours, becoming an important neuroprotection target. Without reperfusion or therapy, penumbra neurons suffer secondary death, expanding the infarct core. This delayed death is mainly driven by excitotoxicity from extracellular glutamate accumulation and N-methyl-D-aspartate receptors (NMDARs) overactivation, followed by massive Ca^2+^ influx and disrupted ion homeostasis. Additional pathological processes include blood–brain barrier (BBB) disruption and neuroinflammation, which exacerbates acute injury [2] but facilitates brain repair processes during recovery [3, 4]. Microglial activation drives transcriptional and morphological changes in close communication with astrocyte via cytokine secretion [5]. Reactive astrogliosis in the peri-infarct region implies morphological transformation, enhanced proliferation/migration, and upregulation of genes such as glial fibrillary acidic protein (GFAP) [6]. Some reactive astrocytes lose neuroprotective functions and acquire a proinflammatory phenotype impairing neuronal and oligodendrocyte survival [7–9]. Within this context, recent data suggest that truncated tropomyosin-related kinase B receptor (TrkB-T1) plays a key role in stroke crosstalk between excitotoxicity, reactive gliosis, and neuroinflammation.

Under physiological conditions, *Ntrk2* alternative splicing produces several TrkB isoforms [10], with full-length TrkB (TrkB-FL) and truncated TrkB-T1 being the major murine cortical variants [11]. TrkB-FL is predominantly neuronal [12], whereas TrkB-T1 is mainly produced by astrocytes and, to a lesser extent, neurons. Binding of brain-derived neurotrophic factor (BDNF) to TrkB-FL activates its tyrosine kinase, triggering pathways promoting neuronal survival and synaptic function. Key downstream mechanisms involve pro-survival transcription factors such as cAMP response element-binding protein (CREB) [13] and myocyte enhancer factor 2 (MEF2) [14, 15], which regulate genes encoding BDNF [16–18], TrkB [19] and specific NMDAR subunits [20]. In neurons, TrkB-T1 lacks the tyrosine kinase domain but binds BDNF, acting as a dominant-negative receptor that inhibits neurotrophic-signalling [21]. TrkB-T1 also exerts TrkB-FL-independent actions, including fine-tuning neurotrophic cascades [22] and regulating astrocyte functions like calcium release [23], neurotransmitter transport [24, 25], and cell maturation/morphology [26, 27]. These independent effects presumably rely on protein interactions via its conserved, isoform-specific C-terminal sequence (FVLFHKIPLDG) [11, 21]. Recently, we isolated the TrkB-T1 isoform-specific interactome, demonstrating interactions with proteins involved in gene expression and mRNA regulation, including splicing and translation [28].

Neurotrophic support becomes aberrant after stroke. Excitotoxicity unbalances TrkB isoforms, decreasing TrkB-FL in the infarct core [29–31] and increasing TrkB-T1 in surrounding astrocytes [32], thus promoting brain injury and oedema [33]. Early TrkB-T1 upregulation occurs alongside increased GFAP expression in the emerging scar, with most GFAP^+^ astrocytes expressing the inflammatory marker complement component 3 d (C3d). Later, GFAP^+^/C3d^+^ cells highly increase alongside macrophage infiltration and microglial activation [28]. Similar TrkB-T1 upregulation occurs in neurodegenerative diseases [31], Down syndrome [34] and spinal cord injury [35], where restoring physiological levels [34] or deleting TrkB-T1 [36] facilitates recovery. We recently demonstrated that TrkB-T1 isoform-specific interactions promote stroke-associated reactive gliosis and neuroinflammation. Specifically, we designed a TrkB-T1-derived cell-penetrating peptide (CPP), TT1_Ct_, combining a BBB-permeable HIV Tat sequence with the TrkB-T1 isoform-specific sequence. TT1_Ct_ reduces reactive gliosis, neuroinflammation, infarct size, and neurological damage [28], suggesting that TrkB-T1 acts as a master regulator of these interconnected pathological processes in stroke and other acute and chronic central nervous system (CNS) diseases involving excitotoxicity [37]. Additional mechanisms contribute to aberrant BDNF/TrkB signalling: (a) retrograde TrkB-FL transport to the Golgi apparatus for calpain processing, producing a truncated receptor similar to TrkB-T1 [29, 38–40], (b) alterations in TrkB-T1 isoform-specific interactome promoting neurotoxicity and astrocyte reactivity [28], and (c) activation of regulated intramembrane proteolysis (RIP), involving sequential processing of TrkB isoforms by metalloproteases, shedding identical extracellular domains (ECDs), and γ-secretase, releasing intracellular domains (ICDs) into the cytoplasm [38]. Excitotoxicity-induced RIP predominantly regulates TrkB-T1 whilst being a secondary processing route for TrkB-FL.

RIP contributes to CNS function in physiological (*e.g*. synaptic function [41]) and pathological conditions [42, 43]. Regarding TrkB, we previously demonstrated that TrkB-ECD acts as a BDNF-scavenger, impairing BDNF/TrkB signalling [38]. However, the role of TrkB-T1-ICD remains uncharacterised. Some γ-secretase-cleaved products from other receptors translocate to the nucleus and modulate gene expression. For instance, Notch-ICD migrates to the nuclei and represses CREB-dependent transcription, affecting learning and memory [44]. In this work, we first characterise TrkB-T1-ICD formation and fate in primary cortical cultures after NMDAR overactivation. We then generate a TrkB-T1-ICD mock CPP for in vitro and in vivo testing. Results reveal that this short intracellular region alone is sufficient to induce TrkB isoform imbalance and skew glial activation toward proinflammatory profiles, resembling important stroke-related pathological mechanisms. Additionally, our data strongly suggest that TrkB-T1 RIP represents a primary mechanism of post-stroke brain injury affecting both neurons and glia.

## Results

### TrkB-T1-ICD formation and nuclear translocation are promoted by NMDAR overactivation in neurons but limited in astrocytes

**Fig. 1:**
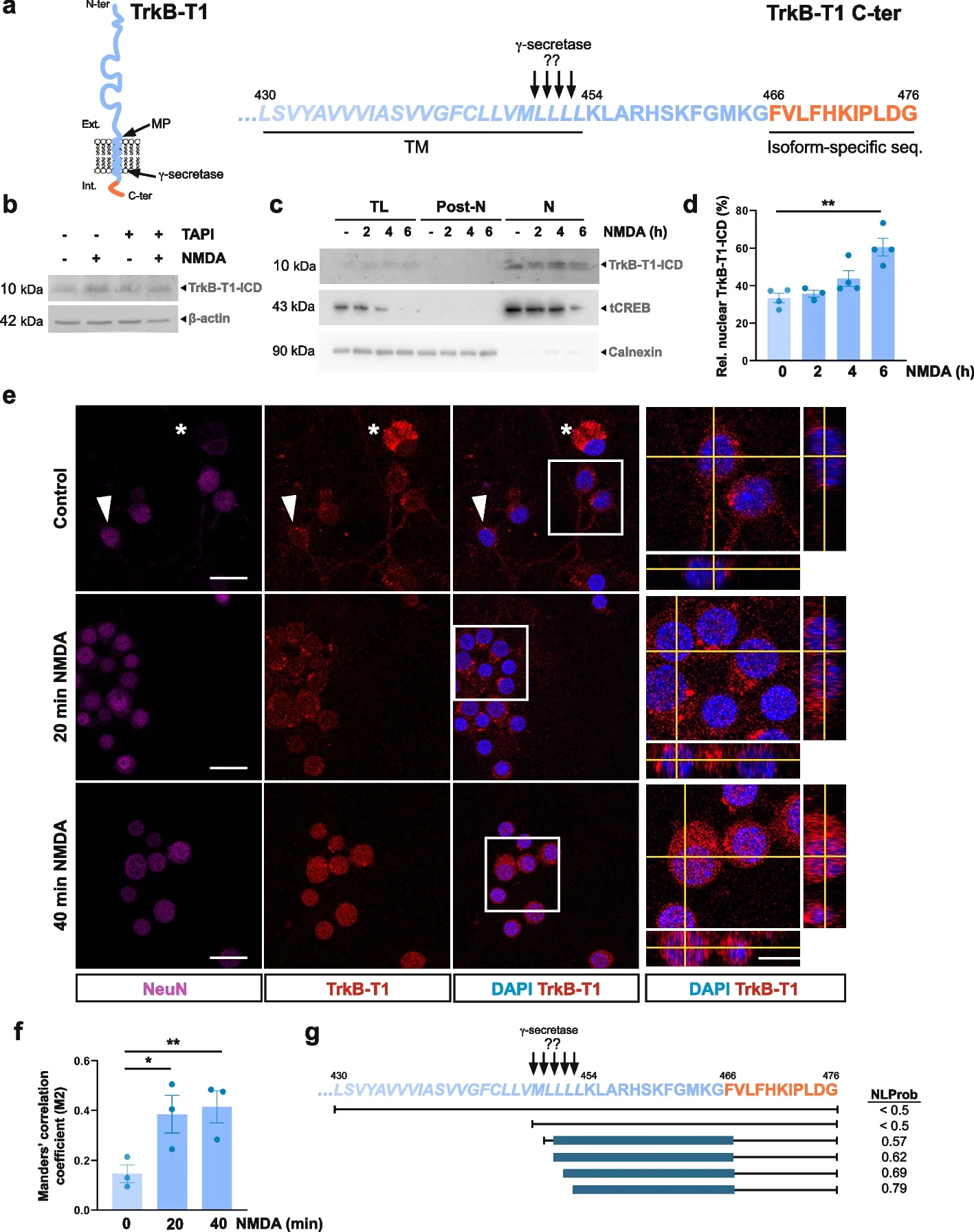
TrkB-T1 RIP induced by excitotoxicity leads to TrkB-T1-ICD formation and nuclear migration in neurons. **a** Scheme of TrkB-T1 membrane orientation with potential sites of metalloproteinase (MP) and γ-secretase cleavage, located in unknown extracellular and intramembrane amino acids, respectively. The TrkB-T1 C-terminal sequence is also shown, with indication of the transmembrane (TM) region (*italics*) and the intracellular sequence, which contains the isoform-specific sequence (orange). **b** Primary cultures of cortical neurons were treated for 6 h with NMDA (100 µM) and glycine (10 µM), herein denoted simply as NMDA. When indicated, cells were preincubated for 30 min with the broad-spectrum metalloprotease inhibitor TAPI-2 (10 µM). A low molecular weight C-terminal fragment, probably TrkB-T1-ICD, was detected in total cell lysates by immunoblot with a TrkB-T1 isoform-specific antibody. The levels of ß-actin were used as a control. **c** Subcellular fractionation of cultured neurons treated for 2, 4 or 6 h with NMDA as before. A low molecular weight TrkB-T1 C-terminal fragment was recognised by the TrkB-T1 isoform-specific antibody in total lysates (TL) and the nuclear fraction (N). Correct cell fractionation was confirmed by using calnexin as a Post-nuclear (Post-N) marker and tCREB as a nuclear (N) marker. **d** Quantification by densitometric analysis of TrkB-T1-ICD nuclear levels. Levels of nuclear TrkB-T1-ICD for each time point were expressed relative to those corresponding to the maximum values obtained for that particular experiment (100%). Individual data and mean ± SEM (*n* = 4) are presented. Results were analysed by one-way ANOVA test followed by post hoc Dunnett’s test. The comparison between the control and 6 h of NMDA treatment is statistically significant (**P = 0.0011). **e** Immunocytochemistry assay of cortical cultures in basal conditions and treated with NMDA for either 20 or 40 min. TrkB-T1 distribution was visualized by labelling with the isoform specific antibody (red). Neurons (arrowheads) were identified using the specific marker NeuN (magenta) and nuclei were stained with DAPI (blue). NeuN^−^/TrkB-T1^+^ cells (asterisk) were rare and probably correspond to a small astrocyte subpopulation present in these cultures. Representative confocal microscopy images are presented. Details of a particular area with data visualization by orthogonal projections are shown in the right panels. Scale bar represents 20 µm in all images. **f** Quantitation of TrkB-T1 staining associated to neuronal nuclei after treatment with NMDA. Mander’s correlation coefficient (M2) between TrkB-T1 and DAPI staining was analysed in confocal microscopy images. Mean ± SEM (*n* = 3) are presented. For each independent experiment, 10 cells/field and 3 technical replicates were analysed. Results were analysed by multiple linear regression model (*P = 0.0117, **P = 0.0054). **g** Analysis using ProtNLS prediction tool of the presence of a nuclear localization signal (NLS, dark blue) in different TrkB-T1-ICDs which might be generated by γ-secretase cleavage in alternative amino acids close to TrkB-T1 transmembrane/cytoplasm boundary. Nuclear localization probability (NLProb) is indicated for each possible TrkB-T1-ICD, values > 0.5 being indicative of a possible nuclear localization

The precise TrkB-T1 intramembrane sequence cleaved by γ-secretase remains undefined due to the limited amount of TrkB-T1-ICD accumulated in neurons following TrkB-T1 RIP [38]. Initial γ-secretase cleavage depends mostly on substrate structure rather than a specific amino acid sequence, and typically occurs near the transmembrane/cytoplasm boundary [43]. Thus, TrkB-T1-ICD is probably formed by a short stretch of TrkB-T1 transmembrane region followed by the 23 amino acids intracellular C-terminal domain (Fig. 1a). This comprises a sequence shared with TrkB-FL (amino acids 454–465, light blue) and a TrkB-T1-specific sequence (amino acids 466–476, orange). In our studies, we used an antibody targeting this exclusive TrkB-T1 sequence [28], which detects both the complete 95 kDa isoform [38] and its C-terminal fragments. In primary cultures of cortical neurons, overactivation of NMDARs with co-agonists NMDA and glycine (abbreviated here as NMDA) promoted TrkB-T1 RIP and generation of low amounts of a TrkB-T1 C-terminal fragment (Fig. 1b). Activation of metalloproteases, the necessary first RIP step, was required for TrkB-T1 processing, as preincubation with the broad-spectrum metalloprotease inhibitor TAPI-2 before NMDA treatment prevented fragment formation. These results align with in vivo observations in a stroke model where metalloproteinase inhibition before ischaemia prevented the formation in the infarcted regions of a low molecular weight TrkB-T1 fragment [28]. To analyse this fragment intracellular location, we fractionated total lysates (TL) from NMDA-treated neurons at different time points (Fig. 1c). Increasing levels of the TrkB-T1 fragment, likely corresponding to TrkB-T1-ICD, paralleled a decrease in nuclear protein CREB (tCREB), which is associated with excitotoxicity [39]. Nuclear fraction (N) analysis showed that, once RIP of this membrane receptor was induced, the generated TrkB-T1 fragment localised in the nucleus, with levels increasing over time (Fig. 1c), differences becoming statistically significant after 6 h (**P < 0.01 relative to basal conditions, Fig. 1d). Immunofluorescence revealed preferential TrkB-T1 distribution in intracellular compartments of untreated neurons, the main cell subpopulation identified by the Neuronal Nuclei marker (NeuN; arrowheads) (Fig. 1e). The isoform was also found in NeuN^−^ cells (asterisk), corresponding to a minor astrocyte subpopulation with much higher TrkB-T1 levels than neurons [28]. A short NMDA treatment induced the nuclear migration of a polypeptide recognized by the TrkB-T1 antibody in NeuN^+^ neurons co-stained with DAPI, a DNA intercalating agent (see selected areas, including orthogonal projections, in the right panels). Quantitation of the spatial overlap showed a significant increase in DAPI and TrkB-T1 co-localization post-treatment compared to basal conditions (Fig. 1f). To investigate the mechanism of TrkB-T1-ICD nuclear translocation, we analysed TrkB-T1 intracellular domain for nuclear localization signals (NLS). ProtNLS, a prediction tool based on pretrained protein models, confirmed the absence of an NLS in full-length TrkB-T1 or a fragment containing the complete transmembrane and intracellular regions (Fig. 1g). However, analysis of putative products produced by standard γ-secretase cleavage [43] unveiled several potential TrkB-T1-ICDs with high nuclear localization probability (NLProb). Downstream of transmembrane residue Met449, four possible TrkB-T1-ICDs contained an NLS (dark blue) that included the intracellular region shared with TrkB-FL. Thus, once generated, these putative TrkB-T1-ICDs may intrinsically possess the ability to migrate to the nucleus.

**Fig. 2:**
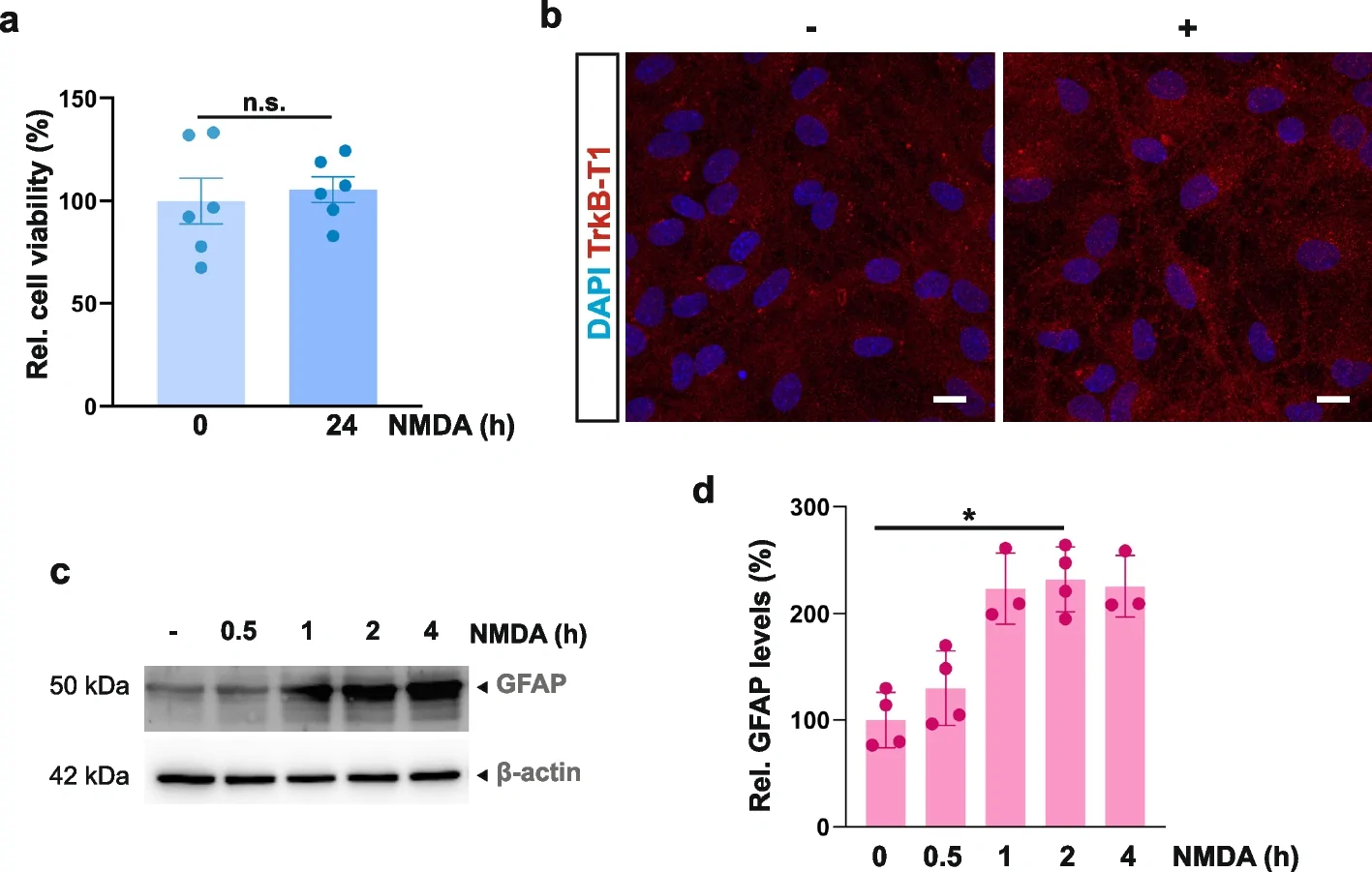
TrkB-T1 RIP is not activated in primary cultures of cortical astrocytes upon NMDA treatment. **a** Cell viability of cultured astrocytes treated with NMDA (400 µM) and glycine (10 µM) for 24 h, herein denoted simply as NMDA. Individual data and mean ± SEM are presented relative to values obtained for untreated cells (*n* = 6). Technical replicates were included in each independent experiment (*N* = 3). Viability levels were analysed by unpaired Student’s *t*-test, being not significantly different (n.s.). **b** Immunocytochemistry assay of cultured astrocytes treated with NMDA for 4 h (indicated as +). Basal conditions are also shown for comparison. TrkB-T1 was detected by immunolabelling with an isoform-specific antibody (red) and cell nuclei using DAPI (blue). Representative confocal microscopy images are shown. Scale bar represents 10 µm. **c** Western Blot analysis of GFAP levels in astrocytes treated with NMDA for 0.5–4 h. β-actin levels were measured as loading control. **d** Quantification by densitometric analysis of GFAP levels. After normalization to β-actin expression, GFAP levels were expressed relative to those corresponding to basal conditions (assigned value of 100). Data was analysed by two-way ANOVA test followed by post hoc Dunnet’s test. Individual values are presented and data are expressed as the mean ± SEM (*n* = 3–4)

NMDARs are also expressed by glial cells, particularly astrocytes, although their function remains poorly understood [45]. Therefore, we investigated if overactivation of astrocytic NMDARs likewise induced TrkB-T1 RIP. We prepared primary cultures of cortical astrocytes, where TrkB-T1 levels are 7 times higher than in neuronal cultures [28]. High NMDA concentrations in these astrocyte-enriched cultures did not affect viability even after 24 h (Fig. 2a), as previously described [46, 47]. In contrast, this glutamate analogue triggered intracellular cascades that increased levels of TrkB-T1 (Fig. 2b) and GFAP (Fig. 2c, d). Nonetheless, we could not demonstrate the formation of either TrkB-ECD or TrkB-T1-ICD upon NMDA treatment (data not shown). Only direct, and probably more extensive, activation of matrix metalloproteinases by p-aminophenylmercuric acetate (APMA) induced a low molecular weight TrkB-T1 C-terminal fragment at very low levels that concentrated in the nucleus (Fig. S1). Based on these results, we propose that glutamate-induced TrkB-T1 RIP is limited in astrocyte monocultures while being strongly induced in neurons exposed to excitotoxicity. Finally, as a preliminary approach to establish a possible contribution of TrkB-T1-ICD to neuronal death, we investigated if generic RIP inhibition was neuroprotective. Cultured neurons were pretreated with TAPI-2 before NMDA treatment (Fig. S2). RIP inhibition had a statistically significant effect on neuronal survival under excitotoxic conditions, increasing neuronal viability in cultures treated with TAPI-2 + NMDA compared to NMDA alone (respectively. 77 ± 2% and 54 ± 12% for 2 h of treatment, *P < 0.05). Nevertheless, attribution of these results solely to reduced TrkB-T1-ICD levels is not straightforward because NMDA-induced RIP also produces TrkB-ECD, a fragment shown to damage BDNF/TrkB signalling [38], and fragments derived from additional potential substrates which might similarly contribute to excitotoxicity.

### A TrkB-T1-ICD mock peptide has specific effects in basal viability of cortical neurons and astrocytes

**Fig. 3:**
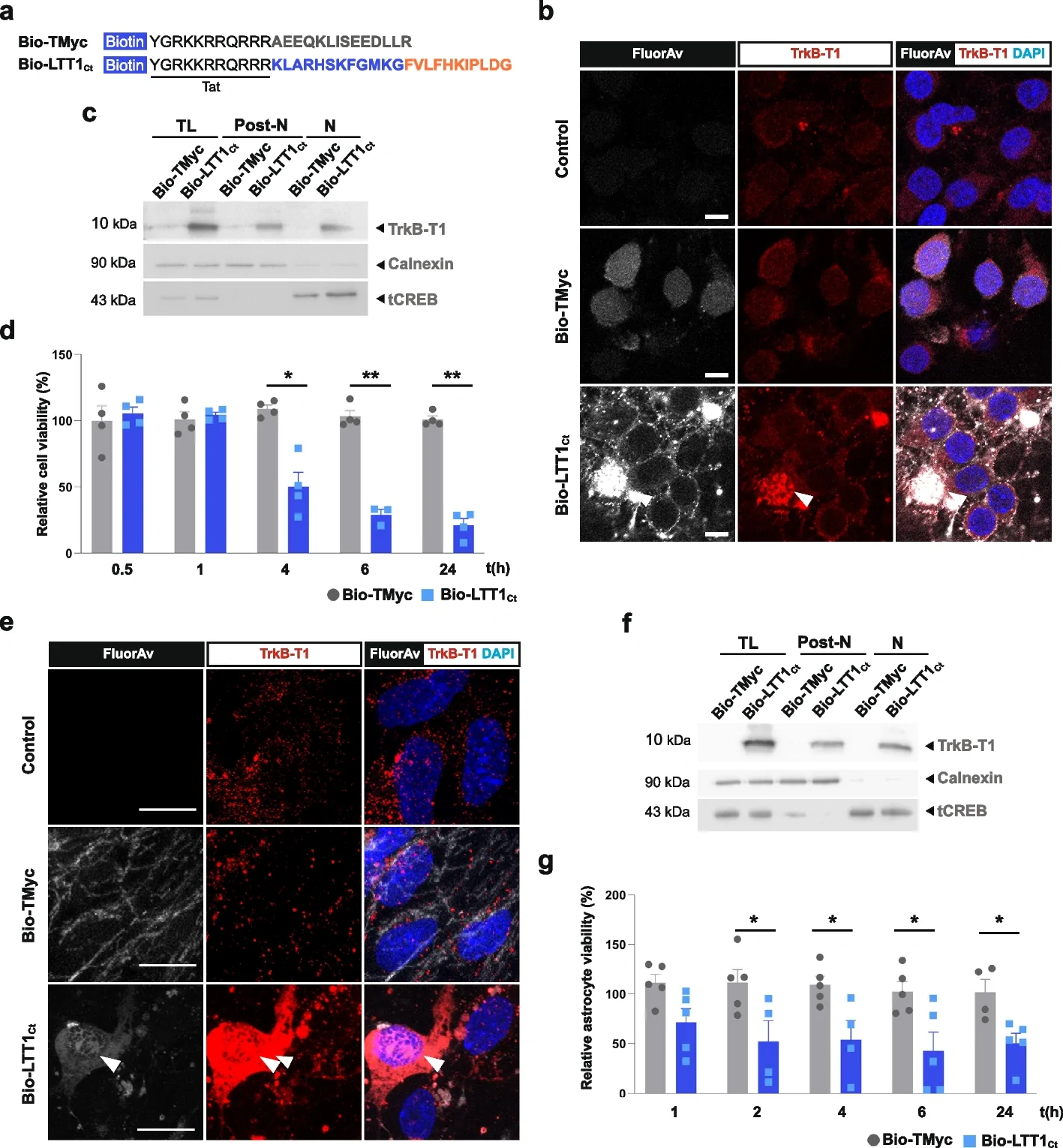
Peptide Bio-LTT1_Ct_ induces cytotoxicity and presents a subcellular distribution similar to endogenous TrkB-T1-ICD. **a** TrkB-T1-derived Bio-LTT1_Ct_ CPP and control peptide Bio-TMyc share a biotin group at the N-terminal, followed by the indicated Tat sequence. After that, Bio-TMyc contains amino acids 408 to 421 of the c-Myc protein (grey), and Bio-LTT1_Ct_ includes TrkB-T1 intracellular amino acids 454–476. Some of these amino acids are common to other TrkB isoforms (amino acids 454–465, dark blue), and others are isoform-specific (amino acids 466–476, orange). **b, e** Immunocytochemistry assays of primary neurons (**b**) and astrocytes (**e**) treated with either Bio-LTT1_Ct_ or Bio-TMyc (25 µM) for 30 min or left untreated (control). TrkB-T1 and Bio-LTT1_Ct_ distribution was examined by labelling with the isoform-specific antibody mentioned above (red). Peptides were visualized with Fluorescein Avidin (FluorAv, white). Nuclear location of Bio-LTT1_Ct_ is indicated with white arrowheads. Representative confocal microscopy images are presented. Scale bars represent 10 µm. **c, f** Subcellular fractionation of primary neurons (**c**) and astrocytes (**f**) treated with either Bio-LTT1_Ct_ or Bio-TMyc (25 µM) for 30 min. Calnexin was used as a Post-nuclear (Post-N) marker and tCREB as a marker of the nuclear (N) phase. Bio-LTT1_Ct_ was detected using the TrkB-T1 isoform-specific antibody. **d, g** Cell viability of primary neurons (**d**) and astrocytes (**g**) treated with Bio-LTT1_Ct_ or Bio-TMyc (25 µM) for selected times. Values are expressed relative to the viability obtained in control cells without peptide (considered as 100%). Individual values are presented and data are expressed as the mean ± SEM. Statistical analysis was performed by two-way ANOVA test followed by post hoc Tukey’s test (**d**, *n* = 4) or Sídak’s test (**g**, *n* = 4–5). We included technical replicates in every independent experiment (*N* = 3)

The challenge of isolating the specific contribution of TrkB-T1-ICD to excitotoxicity led us to perform gain-of-function studies using a CPP mimicking this RIP fragment, Bio-LTT1_Ct_. Because the exact N-terminal amino acid of TrkB-T1-ICD is unknown (Fig. 1a), we excluded the short transmembrane sequence. Thus, Bio-LTT1_Ct_ contains an N-terminal biotin molecule for visualization, an HIV-1 Tat domain enabling attached cargoes to cross the BBB and plasma membranes [48, 49], and the TrkB-T1 intracellular sequence (Fig. 3a). An unrelated c-Myc sequence peptide (Bio-TMyc) served as a negative control. Previous studies have consistently shown Bio-TMyc entry in neurons and astrocytes without affecting viability under basal or excitotoxic conditions [28, 39, 50]. We chose to use this well-characterised control over a scrambled Bio-LTT1_Ct_ version to avoid potential novel structures or unpredicted properties. Immunofluorescence showed neuronal uptake of both Bio-TMyc and Bio-LTT1_Ct_ within 30 min, each peptide displaying a characteristic distribution (Fig. 3b). In Bio-LTT1_Ct_-treated cells, isoform-specific antibody staining revealed a particulate distribution resembling endogenous TrkB-T1, suggesting Bio-LTT1_Ct_ localization near its corresponding receptor. Furthermore, a Bio-LTT1_Ct_ fraction was detected in neuronal nuclei (Fig. 3b, white arrowheads), a result confirmed by cell fractionation (Fig. 3c) that mirrors TrkB-T1-ICD behaviour. Regarding viability, Bio-LTT1_Ct_ (25 µM) was not toxic initially but reduced total cell viability (neurons + astrocytes) to 50 ± 11% after 4 h, significantly lower than control Bio-TMyc (109 ± 3%, *P < 0.05; Fig. 3d). This toxicity was dose-dependent and observed at concentrations > 5 µM (Fig. S3). Similar results were obtained in primary astrocyte cultures regarding peptide entry and distribution (Fig. 3e). Bio-LTT1_Ct_ dissemination resembled TrkB-T1 particulate distribution but was also detected in the nucleus (Fig. 3e, white arrows), consistent with partial migration observed in fractionation experiments (Fig. 3f). Finally, Bio-LTT1_Ct_ was toxic to astrocytes, inducing effects similar to those found in neurons by 4 h at an earlier time point (2 h, *P < 0.05; Fig. 3g). These findings, together with previous results [28], suggest that Bio-LTT1_Ct_ detrimental effects on viability are specific to the TrkB-T1 454–465 sequence (Fig. 3a, dark blue), which we propose acts as an NLS in TrkB-T1-ICD. A shorter peptide lacking these amino acids (Bio-sTT1_Ct_, Fig. S4a) does not affect basal cell viability (Fig. S4b) and, in contrast, is highly neuroprotective in excitotoxicity [28]. Because Bio-LTT1_Ct_ and Bio-sTT1_Ct_ share similar hydrophobicity and charge density, we hypothesise that Bio-LTT1_Ct_ effects on cell viability derive from TrkB-T1 amino acids 454–465, and that this peptide partially recapitulates TrkB-T1-ICD localization, structure and effects, at least in neurons.

### Peptide Bio-LTT1_Ct_ partly mimics excitotoxicity-induced transcriptional changes in neurons

**Fig. 4:**
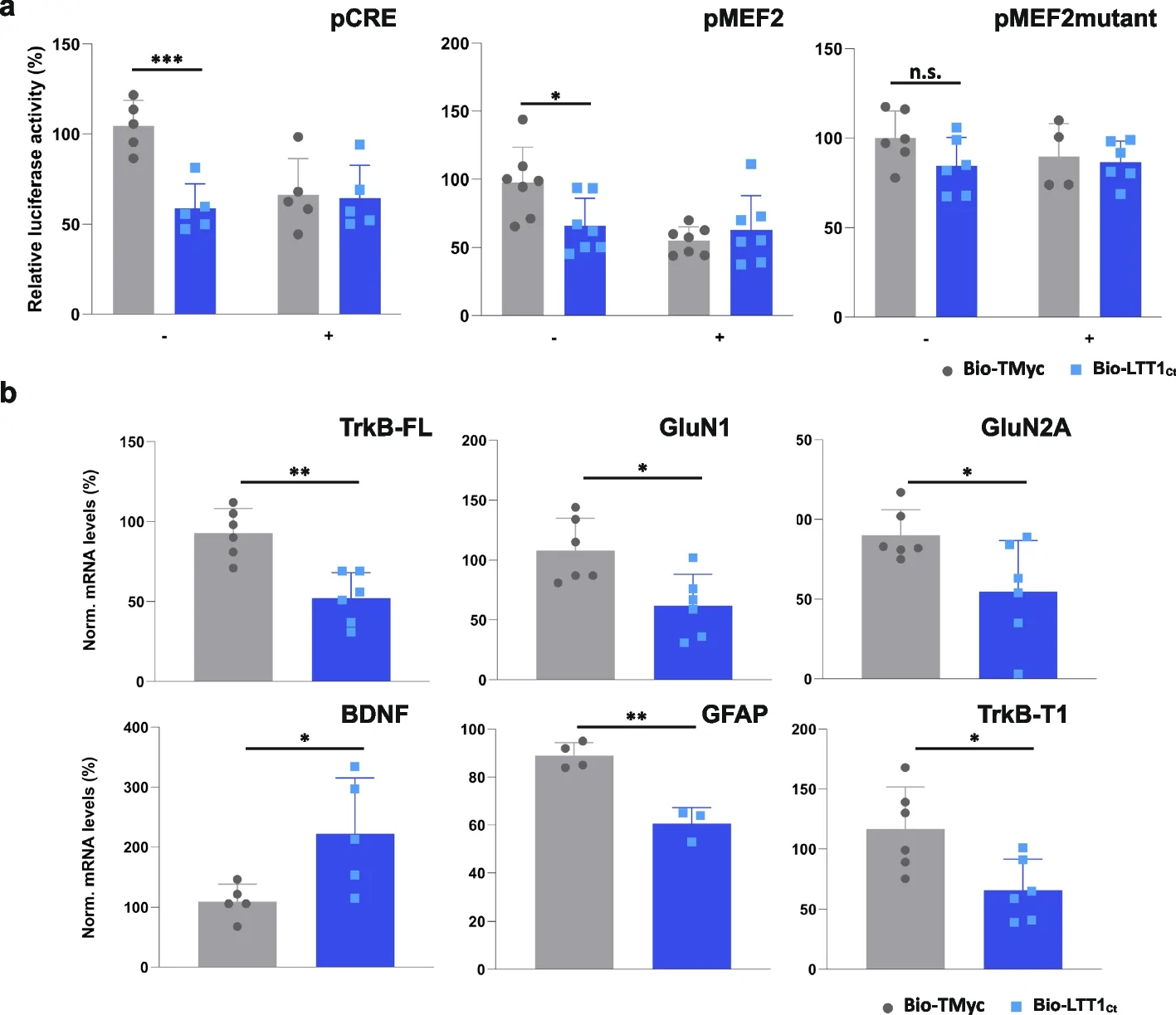
Bio-LTT1_Ct_ effects on transcriptional regulation lead to an excitotoxicity-like scenario. **a** Effect of Bio-LTT1_Ct_ treatment on CRE and MEF2 promoter activities. Cultures transfected for 24 h with luciferase reporter plasmids pCRE, pMEF2 and pMEF2mutant were treated for 2.5 h with either Bio-LTT1_Ct_ or Bio-TMyc (25 µM) (indicated as -). Alternatively, cultures were preincubated with the peptides for 30 min and then subjected to excitotoxicity with NMDA for 2 h, CPPs remaining in the media (indicated as +). Luciferase results are expressed relative to those obtained in untreated cultures. Individual data and mean ± SEM are presented (*n* = 5–7, *N* = 4). Data were analysed by two-way ANOVA test followed by post hoc Sídak’s test. **b** Effect of Bio-LTT1_Ct_ treatment on mRNA levels of CREB/MEF2-regulated genes. Cultures were treated for 2.5 h with either Bio-LTT1_Ct_, Bio-TMyc (25 µM) or left untreated. Levels of mRNA were normalized to neuronal specific enolase (genes mainly expressed in neurons: TrkB-FL, GluN1 and GluN2A) or GAPDH (genes expressed both in neuronal and glial cells: BDNF, GFAP and TrkB-T1). Individual data are presented and expressed as the mean ± SEM of mRNA levels relative to values found in untreated cultures. Data were analysed by Student’s *t*-test (*n* = 3–6, *N* = 3)

The nuclear presence of Bio-LTT1_Ct_ and its effects on neuronal viability suggest that this peptide might mimic excitotoxicity-induced transcriptional changes. NMDAR overactivation downregulates the promoter activity of CREB and MEF2 [39], transcription factors essential for neurotrophic-induced neuronal survival [13, 14] and synaptic activity [51, 52]. Therefore, we performed reporter assays in neurons transfected with pCRE and pMEF2 plasmids, containing promoters with minimal CREB or MEF2 responsive elements controlling luciferase expression [19, 53], and subsequently treated with Bio-LTT1_Ct_, Bio-TMyc or no peptide (Fig. 4a). In pCRE-transfected cells (Fig. 4a, left panel), Bio-LTT1_Ct_ significantly decreased luciferase activity to 59 ± 6% relative to untreated controls, unlike Bio-TMyc (105 ± 6%, *** *P* < 0.001), as described for excitotoxicity [28, 39]. Similar reductions occurred in pMEF2-transfected cells (66 ± 8% *vs*. 98 ± 10% for Bio-TMyc, *P < 0.05; Fig. 4a, central panel). Importantly, Bio-LTT1_Ct_ did not decrease luciferase activity in cells transfected with pMEF2mutant (Fig. 4a, right panel), a plasmid similar to pMEF2 but having two inactivating point mutations in MEF2 responsive elements (Table S1), implying that Bio-LTT1_Ct_ transcriptional effects are a direct mechanism and not secondary to delayed toxicity. Interestingly, the combination of Bio-LTT1_Ct_ and NMDA treatments (Fig. 4a, columns labelled as +) did not exacerbate the inhibition of CREB/MEF2 promoter activities beyond that induced by Bio-LTT1_Ct_ alone, unveiling target convergence within the same pathway.

Next, we analysed mRNA levels of downstream CREB/MEF2-dependent genes to investigate if Bio-LTT1_Ct_ also mimicked excitotoxicity-induced transcriptional changes. Compared to Bio-TMyc, Bio-LTT1_Ct_ significantly reduced mRNA levels of pro-survival neuronal genes such as the *Ntrk2* splicing form encoding TrkB-FL and the GluN1 and GluN2A NMDAR subunits, while increasing *BDNF* mRNA (Fig. 4b). These alterations closely mirror changes described during NMDA-induced excitotoxicity [39, 54]. However, unlike general excitotoxicity [29, 55], Bio-LTT1_Ct_ reduced mRNAs encoding GFAP and TrkB-T1. In conclusion, concurrent with nuclear migration, Bio-LTT1_Ct_ alters CREB/MEF2 promoter activities and the expression of key neuronal survival genes, closely reproducing an excitotoxic environment with some peculiarities that merit further characterisation. Altogether, these findings reveal the importance of TrkB-T1 intracellular domain in regulating CREB/MEF2-dependent transcription in excitotoxic conditions, and suggest that TrkB-T1-ICD might have a relevant role in changes in gene expression induced by excitotoxicity.

### Intranasal administration of Bio-LTT1_Ct_ replicates in cortex stroke-induced expression patterns and astrocyte proinflammatory profiles

**Fig. 5:**
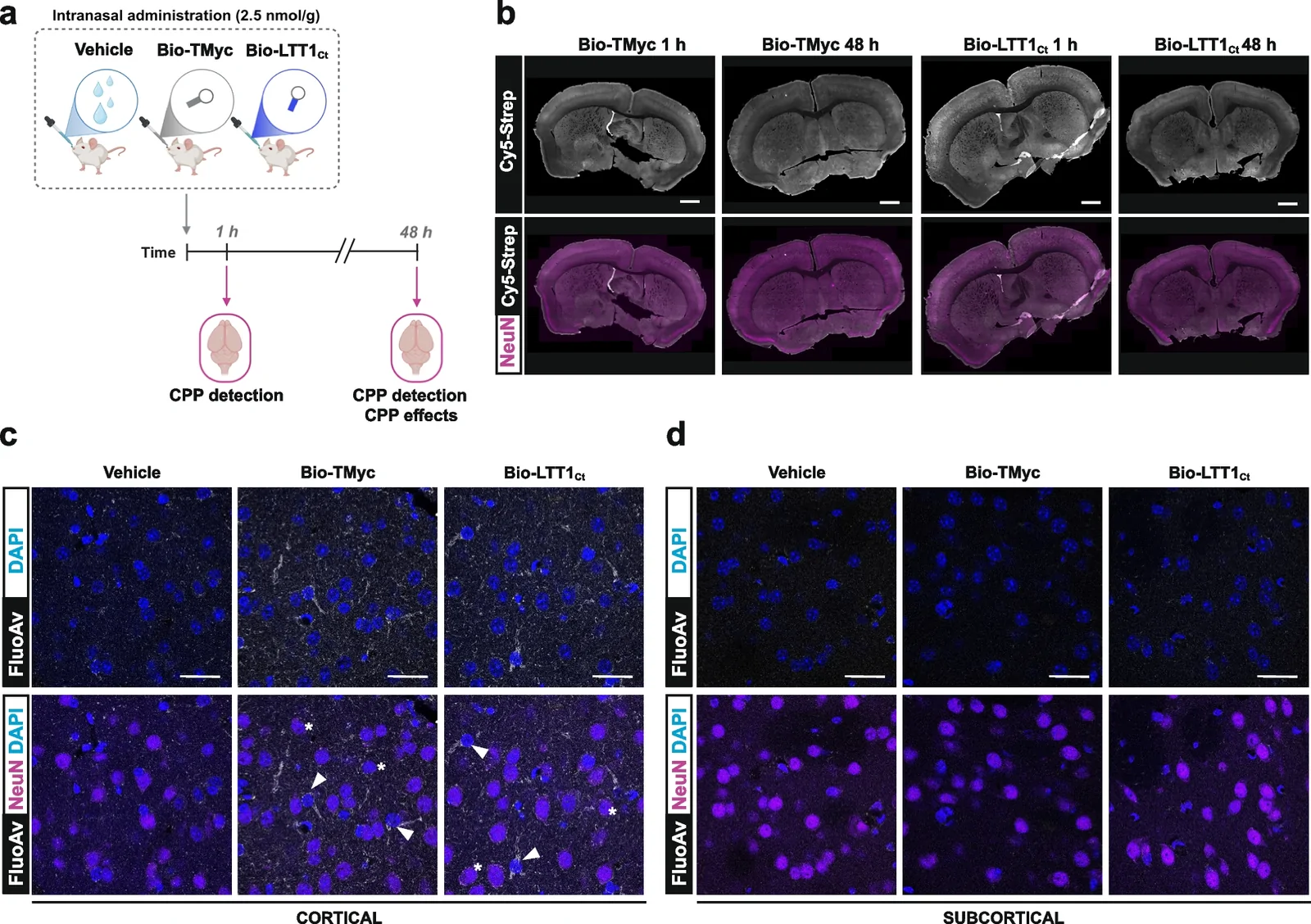
Intranasal (*i.n.*) administration of Bio-LTT1_Ct_ leads to effective brain delivery. **a** Experimental design. Adult male mice receiving a single dose of either vehicle, Bio-TMyc or Bio-LTT1_Ct_ (2.5 nmol/g) by *i.n.* administration were examined 1 or 48 h later, after intracardiac perfusion with 4% paraformaldehyde. Peptide levels were studied both at 1 and 48 h after treatment while the effect of CPPs on brain homeostasis was analysed at 48 h. **b** Peptides were visualized with Cy5-Streptavidin (Cy5-Strep, white) in fixed coronal sections of animals treated with either Bio-TMyc or Bio-LTT1_Ct_ for 1 and 48 h, as indicated. Neurons were also identified using the NeuN antibody (magenta). Wide-field images were obtained with a Cell Observer Microscope. **c, d** Detailed analysis of Bio-LTT1_Ct_ delivery to the mice cortex after 1 h of CPP administration. Bio-LTT1_Ct_ and Bio-TMyc were detected in coronal sections by Fluorescein Avidin D (FluorAv, white), neurons with NeuN (magenta) and cell nuclei with DAPI (blue). We observed peptide delivery in neurons (asterisks) and non-neuronal cells (arrowheads). Representative confocal microscopy images correspond to single sections from cortical and subcortical areas. Scale bars represent 20 μm (*n* = 4 per group)

We next examined if Bio-LTT1_Ct_ could disturb neuron and/or glial function mimicking stroke pathological mechanisms. We used intranasal administration, a non-invasive delivery route to the brain parenchyma that uses trigeminal, olfactory and microvascular pathways to bypass the BBB [56]. Intranasal administration of CPPs is currently under intense investigation to improve brain drug delivery feasibility [57, 58]. Mice were treated with Bio-LTT1_Ct_ or Bio-TMyc for 1 or 48 h, using the vehicle as an additional control (Fig. 5a). We analysed brain delivery and peptide stability via wide-field imaging, using Cy5-Streptavidin (Cy5-Strep, white) for CPP labelling and NeuN immunohistochemistry (magenta) (Fig. 5b). One-hour post-administration, both CPP-treated mice showed similar peptide levels mainly within both cortical hemispheres, regardless of the administration nostril. Peptide presence decreased to background levels 48 h post-administration. Using confocal microscopy, we investigated the distribution of Bio-TMyc and Bio-LTT1_Ct_ in cortical and subcortical brain areas 1 h post-treatment (Fig. 5c, d). Compared to vehicle controls, similar levels of Bio-TMyc and Bio-LTT1_Ct_ were detected in most cortical cells, localised in the cell bodies and projections of both NeuN^+^ (asterisks) and NeuN^−^ cells (arrowheads) (Fig. 5c). Conversely, these biotin-labelled peptides were undetected in subcortical areas of these coronal sections (Fig. 5d). These results demonstrate efficient nose-to-brain delivery of Bio-LTT1_Ct_ and Bio-TMyc, which crossed the BBB of undamaged animals to reach the cerebral cortex. Furthermore, peptide clearance and/or limited tissue stability provide a clear temporal frame to analyse peptide distribution and subsequent brain effects.

**Fig. 6:**
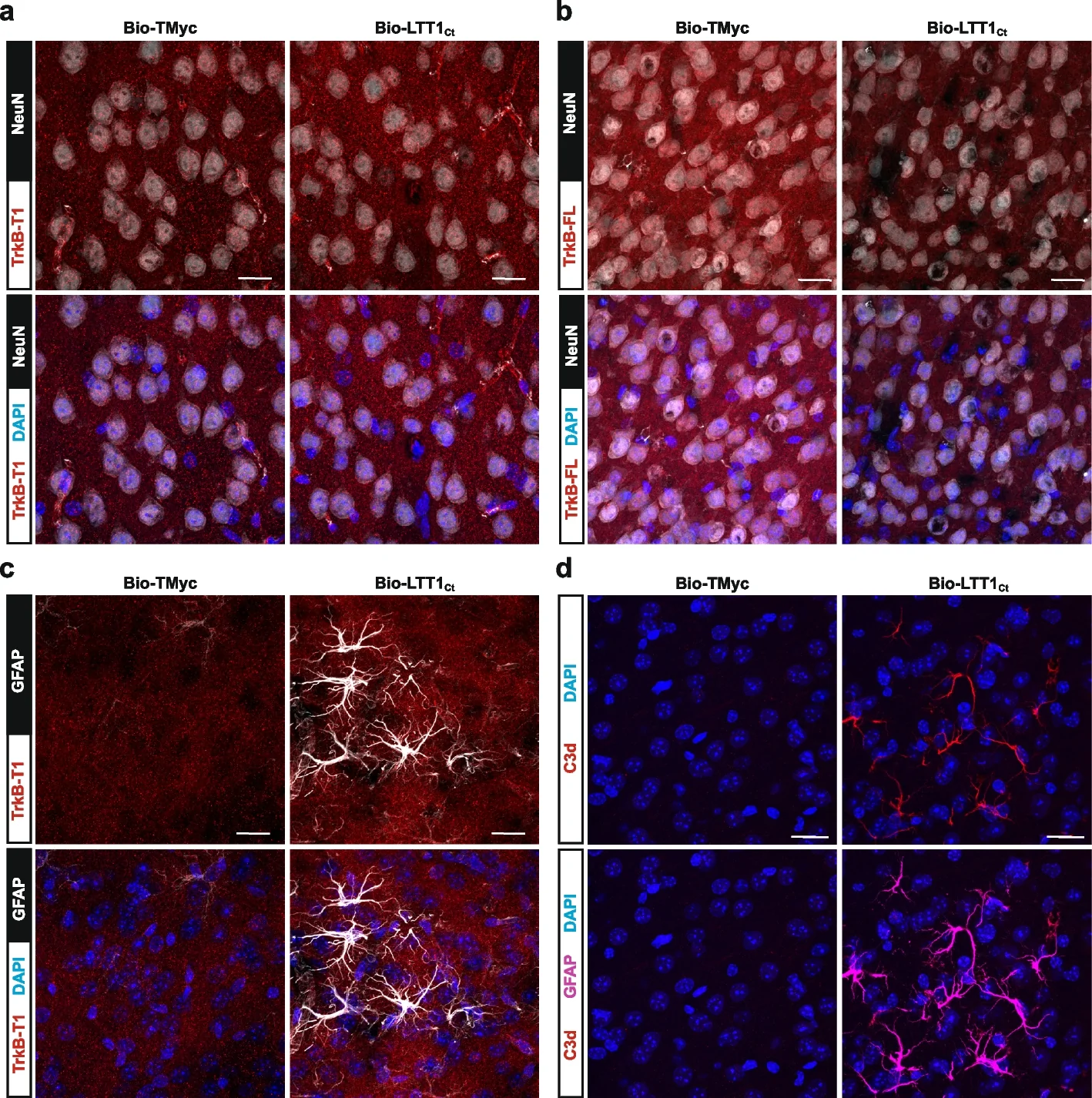
Bio-LTT1_Ct_ induces stroke-like changes in neurons and astrocytes after intranasal administration in vivo. **a, b** Double immunohistochemistry of TrkB-T1 (**a**) and TrkB-FL (**b**) in male mice cortex 48 h after CPP administration. Both TrkB isoforms were detected with isoform-specific antibodies (red), counterstained with NeuN (white) for neurons and DAPI (blue) for nuclei staining. **c** Double immunohistochemistry of TrkB-T1 (red) and GFAP (white) in male mice cortex 48 h after CPP administration. DAPI (blue) for nuclei staining is also shown. **d** Double immunohistochemistry of complement protein C3d (red) and GFAP (magenta) in male mice cortex 48 h after CPP administration. DAPI (blue) for nuclei staining is also shown. Representative confocal microscopy images of cortical areas correspond to maximum intensity projections. Scale bars represent 10 μm (**a, b**) and 20 μm (**c, d**) (*n* = 4 per group)

Previous work demonstrated that ischaemic stroke induces an early decrease of TrkB-FL levels in neurons of the infarcted area and an increase of TrkB-T1 [29], alongside augmented expression of TrkB-T1, GFAP and C3d in scar astrocytes [28]. We hypothesised that these actions were promoted by TrkB-T1-ICD produced in the infarcted tissue as a result of stroke-induced excitotoxicity-dependent TrkB-T1 RIP [38]. To test this hypothesis, we analysed the effect of Bio-LTT1_Ct_ on cortical TrkB isoforms 48 h post-administration (Fig. 6a, b). Compared to Bio-TMyc, Bio-LTT1_Ct_ increased TrkB-T1 (Fig. 6a) in both cortical neurons and astrocytes and decreased neuronal TrkB-FL (Fig. 6b). This native TrkB-T1 analysis was possible because Bio-LTT1_Ct_ becomes undetectable in the cortex by 48 h (Fig. 5b), avoiding antibody interference. Concurrent with TrkB-T1 upregulation, Bio-LTT1_Ct_ increased GFAP levels in cortical astrocytes (Fig. 6c) and promoted a proinflammatory profile, indicated by a strong GFAP/C3d co-staining (Fig. 6d). Altogether, these results demonstrate that Bio-LTT1_Ct_ alone can replicate in vivo specific stroke-induced protein expression patterns in neurons and astrocytes of non-ischaemic animals.

### Intranasal administration of Bio-LTT1_Ct_ promotes a proinflammatory microglial activation state comparable to brain ischaemia

**Fig. 7:**
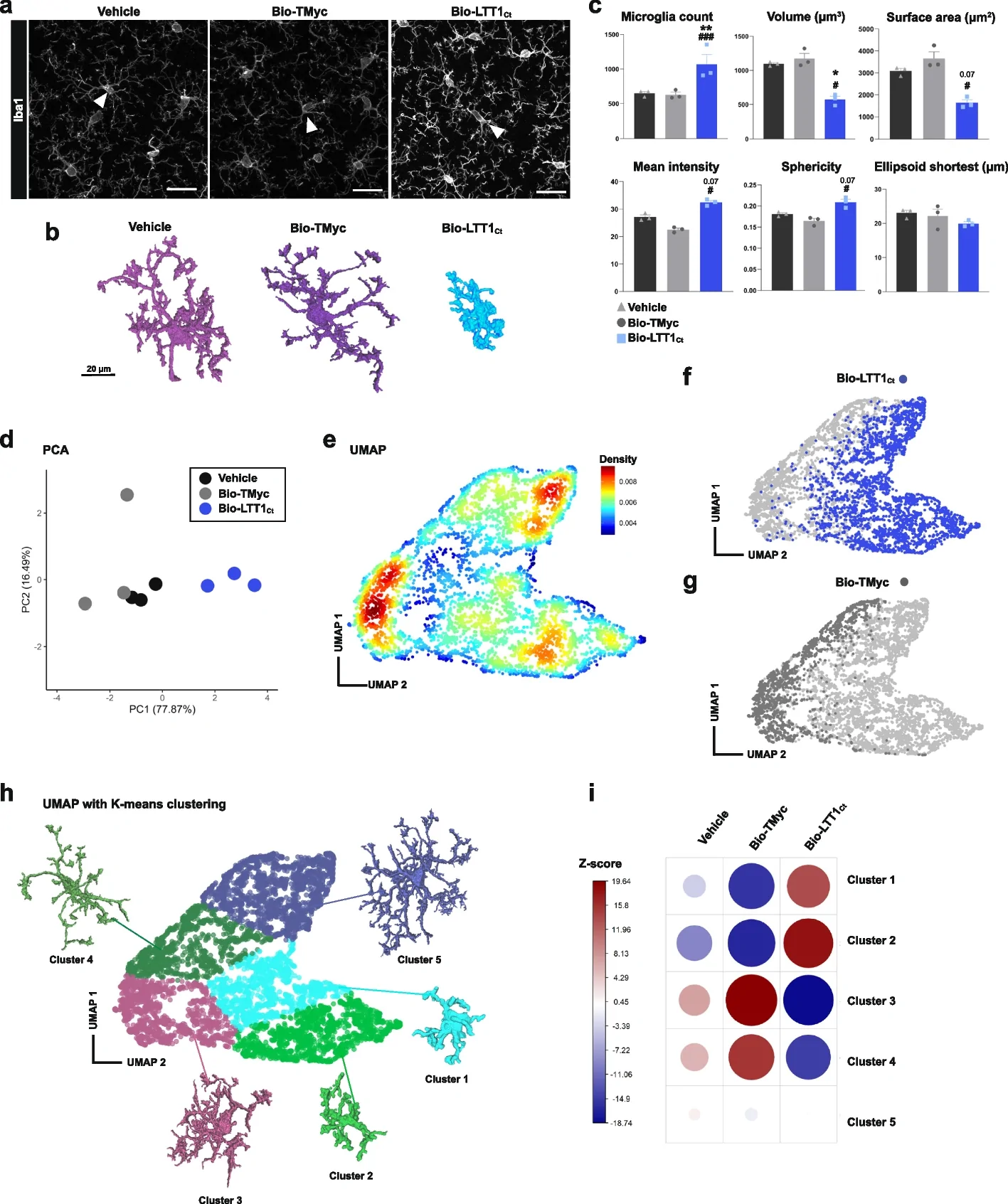
Bio-LTT1_Ct_ promotes microglia activation after intranasal administration in vivo. **a** Immunohistochemistry of Iba1 after 48 h of peptide treatment. Representative images from selected areas of tiles obtained by confocal microscopy from cortical areas are shown (see Fig. S5). Images correspond to maximum intensity projections. Scale bars represent 20 μm. **b** 3D representation of selected cells from the images shown in **a**. Morphological reconstruction was obtained from 3D segmentation of each Iba1 positive cell in the original images. **c** Bar plots represent the individual mean value of selected morphological parameters and number of microglial cells for each animal and treatment. Statistical analysis was carried out by using two-way ANOVA test followed by Tukey’s multiple comparison test (* for vehicle *vs* Bio-LTT1_Ct_, # for Bio-TMyc *vs* Bio-LTT1_Ct_). Data are expressed as the mean ± SEM (*n* = 3 per group, *N* ≈ 1000). **d** PCA representation based on the parameters shown in **c** for animals treated with vehicle, Bio-TMyc or Bio-LTT1_Ct_. Variance represented by the First (PC1) and Second (PC2) Principal Components are shown. **e** UMAP density plot representation of microglial cells from all mice and groups of treatment. Cell density is presented using UMAP1 and UMAP2 dimensions. **f, g** Cells corresponding to either Bio-LTT1_Ct_ (dark blue) or Bio-TMyc (grey) treated animals are respectively highlighted. **h** UMAP representation of unsupervised K-means clustering based on parameters previously indicated. Clusters detected are represented in the same colours as their representative morphological reconstruction. **i** Correlation plot between treatment conditions and presence of the identified morphological clusters. Z-score is presented, red indicating enrichment and blue indicating depletion

**Fig. 8:**
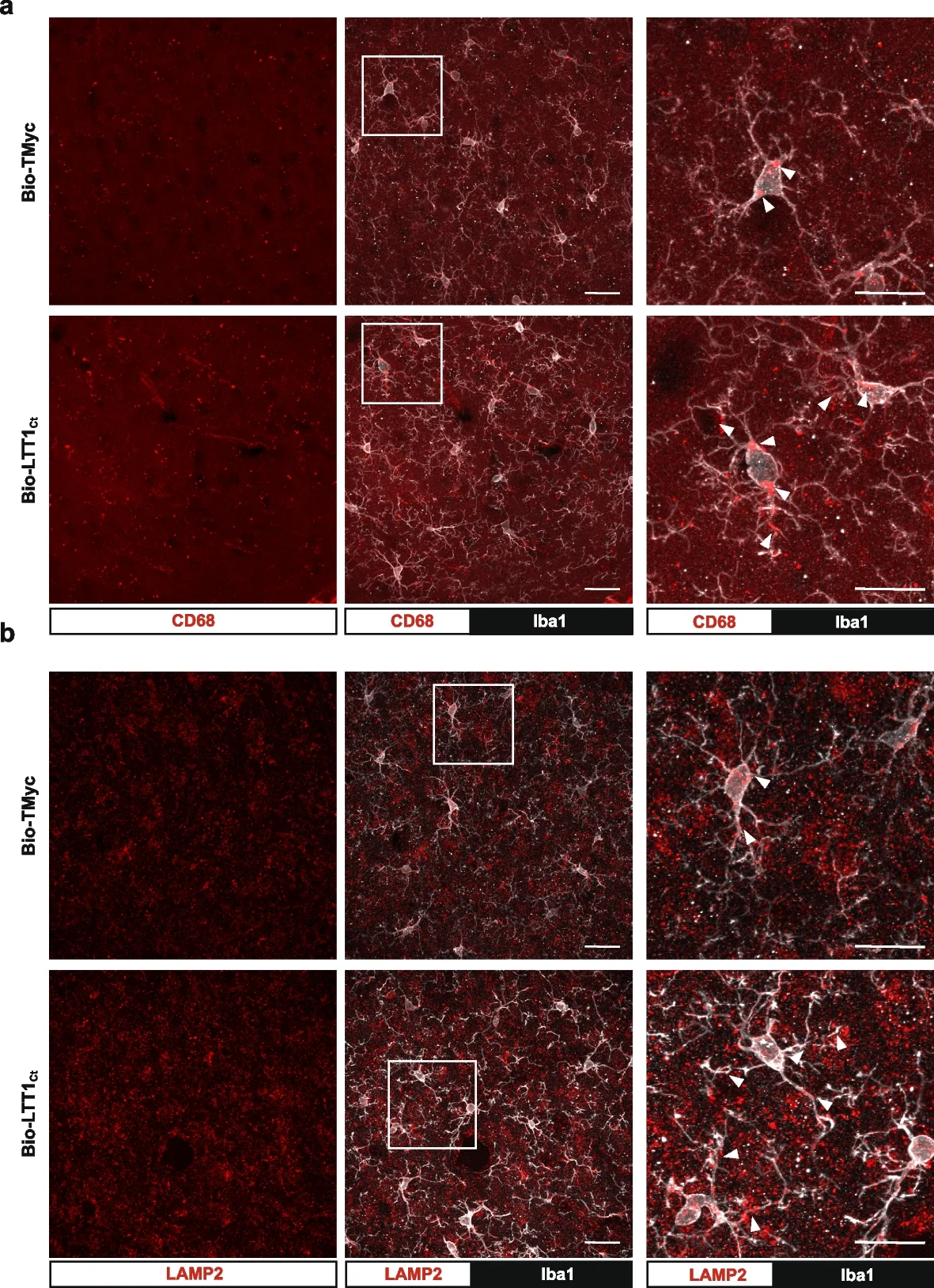
Bio-LTT1_Ct_ treatment triggers microglial proinflammatory reactivity. Immunofluorescence of Iba1 (white) and CD68 (red) (**a**) and Iba1 (white) and LAMP2 (red) (**b**) after 48 h of peptide treatment. Representative confocal images from the cortical areas of Bio-TMyc and Bio-LTT1_Ct_-treated animals are presented (*n* = 3 per group). Scale bars represent 20 μm

Finally, we explored the effect of Bio-LTT1_Ct_ administration on microglia activation (Fig. 7). In the ischaemic brain, microglia display diverse morphological configurations including ramified or amoeboid shapes, corresponding respectively to surveillant or activated microglia [59, 60]. Observation of ionized calcium-binding adapter molecule 1 (Iba1) in the cortex of animals treated with Bio-LTT1_Ct_ for 48 h, revealed morphological differences and a strong increase in Iba1 expression compared to vehicle and Bio-TMyc-treated animals (Fig. 7a, Fig. S5). To characterise these differences, we performed tridimensional reconstructions from Iba1-stained cortical tiles, followed by cell segmentation and measurement of different morphological parameters (volume, area, sphericity, and shortest ellipsoid), cell counts, and mean Iba1 intensity (Fig. 7b, c). Comparison of 3D reconstructed cells showed more rounded and ameboid-like morphological characteristics for microglia in Bio-LTT1_Ct_-treated animals (Fig. 7b), correlating with significantly increased sphericity, and decreased volume and area compared to Bio-TMyc animals (Fig. 7c). Bio-LTT1_Ct_ also increased microglial count and mean Iba1 intensity. Conversely, control peptide Bio-TMyc had no effect on morphology or count, yielding results similar to vehicle (Fig. 7b, c, Fig. S5). Principal Component Analysis (PCA) using these parameters showed clear separation between Bio-LTT1_Ct_ and control groups, indicating a distinct peptide effect on cortical microglia (Fig. 7d). Uniform Manifold Approximation (UMAP) analysis of all Iba1^+^ cells according to above parameters (Fig. 7e) confirmed that Bio-LTT1_Ct_-treated cells (Fig. 7f) showed an opposite behaviour to Bio-TMyc (Fig. 7g) or vehicle groups (Fig. S6). To define morphological subtypes, unsupervised K-means clustering differentiated five groups, visualized in UMAP alongside 3D reconstructions (Fig. 7h). This diversity is expected given microglia heterogenicity and dynamic nature. Correlation analysis confirmed that Bio-LTT1_Ct_ increased rounded, activated-like microglia and decreased resting-like groups (Fig. 7i), whereas control groups remained enriched in resting-like microglia. To assess whether this morphological activation corresponded to a proinflammatory phenotype, we performed double immunofluorescence for Iba1 and either CD68 (Fig. 8a) or lysosome-associated membrane protein 2 (LAMP2, Fig. 8b). CD68, expressed in macrophages, activated microglia and other monocyte-lineage cell types, showed moderate expression in Bio-TMyc controls but increased after Bio-LTT1_Ct_ administration, as for Iba1. These changes resembled those differentially induced by ischaemia in a stroke model (Fig. S7a), where CD68 and Iba1 increased near the infarct in the ipsilateral hemisphere compared to the contralateral region. Additionally, LAMP2, overexpressed in proinflammatory microglia, increased in Iba1^+^ cells following Bio-LTT1_Ct_ treatment (Fig. 8b), mirroring results near the infarct in ischaemia (Fig. S7b). Altogether, these changes indicate that Bio-LTT1_Ct_ promotes a proinflammatory microglial activation state comparable to ischaemia [61, 62]. We propose that, after stroke, TrkB-T1-ICD triggers transcriptional changes depleting pro-survival proteins and promoting proinflammatory astrocyte and microglia profiles, hallmarks of ischaemic stroke and main contributors to brain damage, which Bio-LTT1_Ct_ mimics even in the absence of stroke.

## Discussion

The results presented in this work support the hypothesis that the central roles played by truncated neurotrophin receptor TrkB-T1 in stroke pathology are largely mediated by its RIP product, TrkB-T1-ICD. This proposition is based on our demonstration that the short intracellular sequence of TrkB-T1 is sufficient to recapitulate key pathological features of stroke, including neurotoxicity, glial reactivity, and neuroinflammation. These findings complement the current understanding of how various excitotoxicity-induced mechanisms act on TrkB isoforms to impair BDNF-signalling. Specifically, NMDAR overactivation inverts *Ntrk2* gene transcriptional pattern in neurons, favouring TrkB-T1 expression over TrkB-FL [29]. This transcriptional depletion is complemented by TrkB-FL retrograde transport to the Golgi apparatus and subsequent calpain-processing [29, 38–40]. This cleavage produces both a truncated membrane receptor similar to TrkB-T1, which may act as an additional dominant-negative receptor, and a 32 kDa cytosolic fragment containing the tyrosine kinase domain that translocates to the nucleus [63]. However, elucidating the contribution of TrkB-T1 to stroke pathology has proven far more complex. While TrkB-T1 upregulation in excitotoxic neurons significantly contributes to neurotoxicity by inhibiting BDNF/TrkB-FL-signalling [21, 29], TrkB-T1 also drives stroke pathology through several TrkB-FL-independent mechanisms in neurons and astrocytes. Firstly, we recently demonstrated that TrkB-T1 co-accumulates with GFAP in activated proinflammatory astrocytes at the infarct border early during ischaemia [28]. This proinflammatory activation is triggered by protein interactions established by the TrkB-T1 isoform-specific sequence under excitotoxic conditions, likewise modulating brain infiltration of immunoglobulins and macrophages, and microglia activation in the infarct core and adjacent tissue [28]. Secondly, astrocytic TrkB-T1 upregulation during ischaemia is also associated to the promotion of cerebral oedema and exacerbated brain damage [33]. This is a critical pathological feature, as oedema is an acute complication of ischaemic stroke frequently associated with poor clinical prognosis [64]. Furthermore, we previously proposed that RIP-generated TrkB-T1 fragments, together with isoform upregulation, strongly contribute to stroke pathology [38]. We demonstrated before that TrkB-T1 shedding releases a receptor ectodomain that diminishes neurotrophic support [38]. Now, in this work, we provide robust new data regarding the intracellular fragment. The results suggest that TrkB-T1-ICD, produced in neurons by γ-secretase intramembrane cleavage of a membrane-tethered C-terminal fragment, translocates to the nucleus. Once there, TrkB-T1-ICD likely modulates the transcription of key genes controlling neuronal function and survival/death decisions that have been described as regulated in excitotoxicity [29, 39, 54].

Nevertheless, the contribution of TrkB-T1 RIP to stroke pathology, particularly in astrocytes, presents some controversies. Published data strongly supports a critical role for γ-secretase activity and RIP signalling in reactive astrocytes during ischaemia, most notably via Notch-1 RIP and Notch-1-ICD generation, which drive peri-infarct astrogliosis, exacerbate brain damage and worsen functional outcomes [65–67]. Furthermore, our own previous work demonstrated that TrkB-T1 RIP occurs in the ischemic brain tissue [38]. Therefore, it is possible that TrkB-T1 RIP takes place in vivo in neurons but also astrocytes. The absence of a robust NMDA-induced TrkB-T1 RIP in astrocyte monocultures can be attributed to two main factors. First, the low levels of functional NMDARs expressed in primary astrocytes compared to neurons might be insufficient to induce a robust metalloproteinase activation in those cells after NMDA treatment, while able to mediate Ca^2+^-waves regulating alternative intracellular processes such as gene expression [45, 68]. Another factor might be the inherent limitations of conventional 2D astrocytic cultures that fail to fully reproduce native astrocyte morphology, transcriptome and function [69, 70]. Indeed, glial homeostasis and activation strongly depend on heterotypic cell–cell contacts and microenvironmental cues present in intact tissue [70], lost in astrocyte monocultures. Nevertheless, other mechanisms could be also considered. For example, TrkB-T1-ICD might be primarily produced in excitotoxic neurons and subsequently transferred to glial cells through extracellular vesicles. As essential mediators of intercellular communication in the brain, extracellular vesicles transport signalling molecules such as RNAs, proteins and lipids. They are released by various CNS cells including neurons, astrocytes, and oligodendrocytes, and participate in key physiological processes (*e.g.,* synaptic plasticity, brain development). Conversely, extracellular vesicles also drive pathological mechanisms and, for neurodegenerative diseases, facilitate spreading of proteins such as β-amyloid, tau, ɑ-synuclein, prions, and TDP-43, aggravating neurodegeneration and accelerating disease progression [71]. In stroke, neuron-derived extracellular vesicles carry distinct miRNAs that modulate microglia and influence stroke progression [72]. However, the crosstalk between neurons and astrocytes via extracellular vesicles in stroke remains largely unexplored. Therefore, determining whether TrkB-T1-ICD serves as an extracellular vesicle cargo promoting proinflammatory responses via intercellular communication represents a highly compelling avenue for future characterization.

To complete our knowledge about the specific contribution of TrkB-T1-ICD to stroke pathology, we developed Bio-LTT1_Ct_, a brain-accessible CPP containing TrkB-T1 intracellular region, the main TrkB-T1-ICD component. CPPs are attracting significant interest due to their novelty and useful properties, including efficient translocation across the BBB, high penetration into diverse cell types, and low toxicity [73, 74]. Beyond acting as delivery systems for therapeutics, CPPs serve as powerful tools for dissecting disease mechanisms, particularly those affecting the CNS. Comparing results obtained for Bio-LTT1_Ct_ with those previously reported for Bio-sTT1_Ct_ [28] suggests that TrkB-T1 amino acids 454–465 are critical for stroke-induced neurotoxicity and neuroinflammation. This sequence represents the final C-terminal amino acids shared by TrkB-FL and TrkB-T1 before divergence, and might constitute an NLS for TrkB-ICD once generated. However, the functional impact of this sequence heavily depends on its specific context: while toxic to cultured neurons and astrocytes when contiguous with TrkB-T1-isoform specific sequences (Bio-LTT1_Ct_), it does not affect basal cell viability when adjacent to TrkB-FL sequences (peptide TFL_457_) and it is actually neuroprotective in excitotoxicity [39]. We propose that TrkB-T1 protein interactions, which are critical for transcriptional regulation and drive neurotoxicity and neuroinflammation, might involve the complete intracellular sequence. However, interfering solely with contacts established by the isoform-specific region is sufficient to prevent these pathological processes [28].

From early treatment times, Bio-LTT1_Ct_ distributes in cultured neurons, astrocytes and mice cortex, promoting effects that mirror excitotoxicity. Thus, Bio-LTT1_Ct_ nuclear migration in primary cultures mimics NMDA-induced translocation of native TrkB-T1-ICD. However, unlike standard excitotoxic conditions, which do not decrease the viability of the small astrocyte subpopulation present in neuronal-enriched cultures [46, 47], Bio-LTT1_Ct_ is lethal to both neurons and astrocytes in vitro. Conversely, the peptide is not lethal in vivo*,* at least 48 h after brain administration. The intact brain microenvironment may temporarily buffer or delay the onset of overt cell death which may ultimately occur at later time points. In contrast, highlighting again the limitations of conventional monocultures [69], primary cultured neurons and astrocytes would be directly exposed to Bio-LTT1_Ct_ without the protection of surrounding support networks, thus showing increased susceptibility to toxic insults. Parallel to nuclear migration and before cell death, Bio-LTT1_Ct_ drives transcriptional changes in neuronal-enriched cultures that closely resemble those induced by NMDA. For example, Bio-LTT1_Ct_ reduces the promoter activity of the pro-survival transcription factors CREB and MEF2 in a manner similar to excitotoxicity. Additionally, the combined treatment with NMDA does not exacerbate the results. Based on these findings, we conclude that TrkB-T1 intracellular region might have a critical role in silencing these survival pathways during excitotoxic injury. It would be interesting to investigate whether the decrease in CREB/MEF2 activity results from direct binding of these transcription factors with TrkB-T1-ICD or via indirect signalling pathways. In accordance with the inhibition of CREB/MEF2 activity, a brief Bio-LTT1_Ct_ treatment alters the mRNA levels of CREB/MEF2-dependent neuronal genes related to survival/death (*e.g.,* TrkB-FL, NMDAR-subunits and BDNF), reproducing excitotoxic changes [39, 54]. Only TrkB-T1 and GFAP mRNA levels do not correspond to those expected for excitotoxicity, suggesting that astrocytes in these mixed cultures may already be profoundly affected by Bio-LTT1_Ct_. In contrast, experiments performed in vivo show that Bio-LTT1_Ct_ successfully reproduces in the cortex protein changes induced by ischaemia, including the imbalance of the TrkB isoforms, upregulation of GFAP and C3d expression in astrocytes, and microglial proinflammatory activation. Future studies will deepen on establishing if Bio-LTT1_Ct_ regulation of *Ntrk2* expression is the primary cause of TrkB isoforms imbalance (*e.g*.*,* mediated by alteration of pre-mRNA splicing dynamics) or it is secondary to neuroinflammation promotion. Finally, Bio-LTT1_Ct_ also promotes important morphological and functional changes in microglia partially resembling the activation pattern typically observed in the early hours after brain ischaemia [28].

This study presents several limitations. Thus, while our gain-of-function results imply that TrkB-T1-ICD regulates in vivo gene transcription across neurons, astrocytes, and microglia, they do not entirely prove that endogenous TrkB-T1-ICD is strictly necessary for the observed pathological effects in stroke. Nevertheless, complementary experiments to achieve TrkB-T1-ICD loss of function in stroke, through RIP inhibition, would be inconclusive due to combined effects with the second TrkB-T1 RIP product, TrkB-ECD. An additional limitation is that Bio-LTT1_Ct_ does not include the complete TrkB-T1-ICD sequence. Difficulties in the isolation and sequencing of neuronal TrkB-T1-ICD have caused a partial ambiguity about its precise amino acid composition. Because γ-secretase typically cleaves substrates close to the transmembrane/cytoplasm boundary [43], TrkB-T1-ICD is expected to contain only 1 to 4 extra leucine residues at the N-terminus of the intracellular sequence. Another apparent limitation is that Bio-TMyc and Bio-LTT1_Ct_ distribution is restricted to the cortex 1 h after intranasal administration. As uptake rates, enzymatic degradation, and clearance kinetics can vary significantly between the cortex and subcortical regions, it remains possible that transient striatal uptake occurs at earlier or intermediate time points (*e.g*., prior to 1 h or between 1 and 48 h) and was not captured here. Regarding peptides’ brain lifespan, we previously demonstrated cortical presence of Bio-TMyc and Bio-TT1_Ct_, a peptide similar to Bio-sTT1_Ct_, at least 4 h after intravenous administration [28]. Independently of residency duration, results suggest that a few hours (< 48 h) are sufficient for Bio-LTT1_Ct_ to promote brain neuroinflammation. Future longitudinal studies are warranted to characterise Bio-LTT1_Ct_ pharmacokinetic properties and distribution across different brain regions, and to evaluate if delivery into deep brain structures needs optimization.

Despite these limitations, our results have relevant clinical implications. Treatment of ischaemic injury requires integrative stroke protection strategies exerted by brain-accessible therapeutic molecules having multimodal and multicellular effects. Building on our previous findings, we propose that receptor TrkB-T1—specifically a small fragment containing its intracellular region, TrkB-T1-ICD, a product of excitotoxicity-induced RIP—has critical roles in neurotoxicity, aberrant neurotrophic-signalling, glial reactivity, and neuroinflammation. This proposition is of pivotal importance because these pathological signatures are linked not only to stroke but also to many other acute and chronic CNS disorders associated with excitotoxicity. Supporting this hypothesis, inflammation, cellular proliferation and migration caused by spinal cord injury are strongly attenuated in TrkB-T1 knockout astrocytes [36]. This genetic modification facilitates functional recovery in this model and reduces neurodegeneration in experimental autoimmune encephalomyelitis [75]. Nevertheless, total TrkB-T1 elimination is not a viable therapeutic strategy since it compromises astrocytes structural integrity, disrupting BBB function and neuronal metabolic support [76]. Consequently, the primary challenge lies in selectively modulating the receptor’s pathological signalling without disturbing its physiological functions. We previously targeted excitotoxicity-induced TrkB-T1 protein interactions promoting stroke-associated reactive astrogliosis and neuroinflammation [76]. To this end, we developed an interfering peptide, TT1_Ct_, which modulates immunoglobulin and macrophage infiltration, alongside astrocyte and microglia activation, ultimately conferring neuroprotection against stroke [28]. However, complementary therapeutic approaches aimed at interfering with TrkB-T1 RIP merit further investigation. Given the contribution of RIP to physiological CNS functioning [41], future therapeutic tools must be engineered to avoid generic RIP inhibition while providing TrkB-T1 site-specific cleavage inhibitors that prevent RIP of this receptor under pathological conditions.

In summary, our findings support TrkB-T1 RIP as a primary mechanism of post-stroke brain injury and demonstrate that the intracellular region of this isoform is sufficient to promote brain glial reactivity, neuroinflammation and neurotoxicity, resembling important stroke-related pathological features.

## Materials and methods

### Primary cortical neurons

Brain cortices of 18-day-old embryos (E18) from Wistar rats, both male and female, were dissected to establish primary cultures. After 7 days in vitro (DIVs), growth of glial cells was repressed, and treatments were started after 12 DIVs. Cultures mainly contained mature neurons and a small proportion of glial cells, mostly astrocytes [29], which helped to sustain non-toxic glutamate concentrations in medium. Robust in vitro excitotoxicity was induced by incubation with high concentrations of glutamate analogue NMDA (100 µM) and co-agonist glycine (10 µM), herein denoted simply as NMDA. This treatment causes neuronal death while astrocyte viability is not affected [29]. Cultures were incubated with TAPI-2 (10 µM) for 30 min before NMDA addition as indicated. Further details (reagents, resources and experimental procedures) are provided as Supplementary material.

### Primary cortical astrocytes

Dissected cortices obtained from Wistar rat E18 embryos as described before were used to prepare primary cultures of differentiated astrocytes, treated 24 h later with NMDA (400 μM) or APMA (100 μM) for selected times. Further details are provided as Supplementary material.

### Model of brain damage by Bio-LTT1_Ct_ administration

Adult male mice were treated with a dose of Bio-TMyc or Bio-LTT1_Ct_ peptides (2.5 nmol/g), administered intranasally into one nostril to mildly sedated animals. Details about CPPs are provided as Supplementary material. Animals were sacrificed 1 or 48 h later and intracardially perfused for immunohistochemistry analysis.

### Model of brain ischaemia by photothrombosis

Permanent ischaemia was induced in adult male mice cortex by microvascular photothrombosis, adapted from [39]. This model implicates injury to the vascular endothelium and platelet activation, provoking microvascular thrombotic occlusion of a brain area selected by a stereotaxic frame. We provoked a focal injury in primary motor and somatosensory cortices using coordinates + 0.2 AP, + 2 ML, relative to Bregma. Further details of the procedure are provided as Supplementary material.

### Assessment of cell injury in cortical cultures

Thiazolyl blue formazan (MTT) reduction assay was used to determine total cell viability after Bio-LTT1_Ct_ (neurons and astrocytes) or NMDA (astrocytes) treatments. Only for analysis of RIP contribution to excitotoxicity, we calculated specific neuronal survival. As mentioned before, neuronal cultures contained a small percentage of glial cells, resistant to the excitotoxic damage [46, 47]. Therefore, we calculated glial cells contribution to the total viability values obtained. More details about this procedure are provided as Supplementary material.

### Subcellular fractionation

Post-nuclear (Post-N) and nuclear (N) phases prepared by cell fractionation were compared to total lysates (TL). Protein concentration of different fractions was established by BCA analysis. Further details are provided as Supplementary material.

### Cell transfection and gene reporter assays

Plasmids contained minimal CREB [19] or MEF2 [53] response elements upstream firefly luciferase reporter gene (respectively, pCRE or pMEF2; details in Table S1). Primary cultures were transfected as detailed in the Supplementary material and incubation proceeded to complete 24 h.

### Extraction of RNA and qPCR assay

We extracted and analysed total RNA as detailed in Supplementary material. Results were normalized to the levels of neuronal specific enolase or glyceraldehyde − 3-phosphate dehydrogenase (GAPDH) genes as indicated.

### Visualization of peptides in primary neurons and immunocytochemistry

Biotin-conjugated peptides Bio-TMyc or Bio-LTT1_Ct_ (25 µM) were added to cells grown on coverslips. After 30 min, treated and untreated control cells were fixed for 30 min with 4% paraformaldehyde in phosphate buffer saline, blocked and permeabilised. Then, cells were incubated with Fluorescein Avidin D, and immunocytochemistry proceeded as described in the Supplementary material.

### Visualization of peptides in brain and immunohistochemistry

After intranasal (*i.n.*) peptide administration for 1 or 48 h, mice were deeply anesthetized and intracardially perfused. The details of brain fixation and cryoprotection for peptide visualization and/or immunohistochemistry are provided in the Supplementary material.

### Statistical analysis

Data are expressed as mean ± standard error of the mean (SEM) of at least three independent experiments in all cases (*n* ≥ 3). For gene reporter, mRNA and viability assays, technical replicates (*N*) were incorporated in every independent experiment. Specific details of the *N* and *n* values, and the statistical test applied in each case can be found in the respective figure legends. The assignment of treatment was performed randomly. Statistical analyses were performed in GraphPad Prism 8.0.2. and RStudio 2024.04.2. Data normality was analysed by Saphiro-Wilk test. Significance was defined as: *P < 0.05, ** *P* < 0.01, *** *P* < 0.001, and **** *P* < 0.0001. A *P*-value larger than 0.05 was considered as non-statistically significant (n.s.).

## Supplementary Information

Supplementary Material 1.

## Data Availability

The data that support the findings of this study, including the segmentation code used for analysis, are available within the article and its supplementary material. Availability of materials does not apply.
