# Supplementary material for: The intracellular region of truncated neurotrophin receptor TrkB-T1 is sufficient to promote brain glial reactivity, neuroinflammation and neurotoxicity
Source: Mol Biomed. 2026 Nov 13;7:247. doi: 10.1186/s43556-026-00650-x (PMC13768517; doi:10.1186/s43556-026-00650-x)
Supplement: Supplementary file 1 [file 43556_2026_650_MOESM1_ESM.pdf]

Supplementary Materials

The intracellular region of truncated neurotrophin receptor TrkB-T1 is sufficient to promote brain glial reactivity, neuroinflammation and neurotoxicity

Lola Ugalde-Triviño[1], María C. Serrano[2] and Margarita Díaz-Guerra[1,*]

[1]Instituto de Investigaciones Biomédicas Sols-Morreale (IIBM), Consejo Superior de Investigaciones Científicas-Universidad Autónoma de Madrid, Madrid 28029, Spain

[2]Instituto de Ciencia de Materiales de Madrid (ICMM), Consejo Superior de Investigaciones Científicas, Madrid 28049, Spain

Contents

- Supplementary materials and methods
- Table S1. List of antibodies, chemicals and recombinant proteins, cell-penetrating peptides, primers, commercial assays, experimental models, recombinant DNAs and software
- Fig. S1. Subcellular fractionation of cultured astrocytes treated with APMA.
- Fig. S2. Excitotoxicity-induced RIP contributes to neuronal death of primary cultures of cortical neurons.
- Fig. S3. Dose response effects of Bio-LTT1$_{Ct}$ on total cell viability.
- Fig. S4. Bio-LTT1$_{Ct}$ toxicity is specific to TrkB-T1 454-465 sequence
- Fig. S5. Microglia morphological analysis.
- Fig. S6. UMAP plot representing microglial cells from all mice and groups of treatment.
- Fig. S7. Microglia proinflammatory profile in an experimental *in vivo* model of stroke.
- Segmentation code used for analysis.

Supplementary materials and methods

Antibodies, chemicals and recombinant proteins, cell-penetrating peptides, primers, commercial assays, experimental models, recombinant DNAs and software are described in Table S1, included as Supplementary material.

Synthetic peptides

Synthetic cell-penetrating peptides (> 95 % purity; GenScript) were used for treatment of primary cortical cultures (25 μM, unless otherwise indicated; *in vitro* studies) and mice (2.5 nmol/g; *in vivo* studies). These CPPs contained a HIV-1 Tat sequence (11 amino acids) linked to a specific TrkB-T1 or c-Myc sequence as indicated (see Table S1). Additionally, these peptides contained a biotin molecule at the N-ter, which helped peptide visualization and stabilization, and an amine group at their C-ter. Peptides were directly added to the culture media for *in vitro* experiments or intranasally administered (*i.n.*) for *in vivo* tests.

Experimental models

Animal procedures were performed following European Union Directive 2010/63/ EU and were approved by the CSIC and *Comunidad de Madrid* (Ref PROEX 276.6/20) ethics committees. The housing facility was approved by *Comunidad de Madrid* (#ES 280790000188) and conforms to official regulations. Animals had standard health and immune status and were looked after by professional caretakers daily. Male mice were kept in groups of up to five in standard IVC cages while 1–2 pregnant rats occupied standard cages, always containing bedding and nesting materials. Animals were under controlled lighting conditions (12 h light/dark cycles), relative humidity and temperature. Irradiated food and water were provided *ad libitum*. The three R's principles were carefully respected.

Mice model of ischaemia by microvascular photothrombosis

This model of permanent focal ischemia mimics embolic or thrombotic occlusion of small arteries, frequently found in human stroke. The procedure was carried out as previously described [1] with some modifications. Male Balb/cOlaHsd mice (23–31 g; 10–16 weeks of age; Janvier Laboratories, France) were allowed to acclimatize for at least 1 week upon reception, being kept with the same cage mates, and daily examined to check their health status. For ischaemic induction, mice were anesthetized with isoflurane (5 % for induction, 2 % for maintenance in oxygen; Abbot Laboratories, Madrid, Spain) and placed in a stereotaxic frame (Narishige Group, Tokyo, Japan), controlling body temperature using a self-regulating heating blanket (Cibertec, Madrid, Spain). A midline scalp incision was made, the skull was exposed, and both Bregma and Lambda points were identified. A cold-light (Schott KL 2500 LCD; Schott Glass, Mainz, Germany) with a fibre optic bundle of 1.5 mm in diameter was centred using a micromanipulator on the right side, at 0.2 mm anterior and 2.0 mm lateral (+0.2 AP, +2 ML) relative to Bregma. Then, the photosensitive dye Rose Bengal (20 mg/kg; Sigma-Aldrich) was intravenously administered and, five minutes later, the brain was illuminated (600 lms, 3,000 K) through the intact

skull for 10 min. Brain injury in this model comprises damage to vascular endothelium, platelet activation, and subsequent microvascular thrombotic occlusion of the irradiated region [2]. The irradiated and, therefore, damaged area corresponds to primary motor and somatosensory cortex (hindlimb and forelimb). Five hours after damage induction, animals were deeply anesthetized and intracardially perfused with cold phosphate buffer saline (PBS) and 4 % paraformaldehyde in PBS, and processed as indicated below.

Primary cultures of rat cortical neurons to study *in vitro* excitotoxicity

The dissected cerebral cortices of Wistar rat embryos (E18), both genders included, were mechanically dissociated in culture medium (Minimum Essential Medium, MEM, Life Technologies) followed by seeding of the cell suspension at a density of 1.1 x 10^6 cells/ml, prepared in the same medium supplemented with 22.2 mM glucose, 0.1 mM glutamax, 5 % foetal bovine serum (FBS), 5 % donor horse serum (HS), 100 U/ml penicillin and 100 µg/ml streptomycin. Before seeding, the plates were treated overnight with 100 µg/ml poly-L-lysine and 4 µg/ml laminin at 37°C. After seeding, the cells were incubated in an atmosphere of 5 % CO_2 and 95 % humidity at 37 °C. In these cultures, glial growth was inhibited after 7 days *in vitro* (DIVs) by adding 10 µM 5 cytosine β-D-arabinofuranoside (AraC) and experimental treatments took place after 12 DIVs. Excitotoxicity was induced by incubation with NMDA (100 µM) and glycine (10 µM) for the indicated times, herein abbreviated as NMDA. As indicated, cultures were preincubated with Tat-derived CPPs before NMDA treatment.

Primary cultures of rat cortical astrocytes and treatment

Dissected cerebral cortices obtained from Wistar rat embryos (E18) as described before were mechanically dissociated in Hank's Balanced Salt Solution (HBSS, Thermo Fisher) containing $CaCl_2$ and $MgCl_2$, supplemented with 10 mM HEPES. The resulting cell suspension was then seeded in 75 cm² flasks pretreated with 100 µg/mL poly-L-lysine at a density of 4 x 10^6 cells/ml in proliferation medium. This medium consisted of Dulbecco's Modified Eagle Medium/Nutrient Mixture F-12 with GlutaMAX (DMEM/F-12/GlutaMAX, Thermo Fisher), 10 % foetal bovine serum (FBS), 5 % horse serum (HS), 100 U/ml penicillin, 100 µg/ml streptomycin, 2 % B27 supplement, 10 ng/ml epidermal growth factor (EGF), and 10 ng/ml basic fibroblast growth factor (bFGF). After 4 DIVs, when cultures reached approximately 70–80 % confluence, the medium was removed, and the flasks were vigorously washed with PBS to eliminate contaminating oligodendrocytes. Cell cultures were then trypsinised using TrypLE Express (Thermo Fisher) and reseeded in flasks under the same culture conditions at a density of 3 x 10^6 cells/ml in proliferation medium. Six days later, astrocytes were washed, trypsinised as before, and seeded at a density of 7 x 10^5 cells/ml in poly-L-lysine-coated plates for 24 h. After this period, the plates were washed with PBS, and the medium was replaced with differentiation medium consisting of DMEM/F-12/GlutaMAX, 10 % FBS, 100 U/ml penicillin, and 100 µg/ml streptomycin. Twenty-four hours later, cultures were treated with 400 µM NMDA or 100 µM APMA for selected times.

Assessment of cell injury in cortical cultures

Thiazolyl blue formazan (MTT) reduction assay was used to determine cell viability. At corresponding time points, 0.5 mg/ml MTT was added to the medium and incubated for 2 h at 37 ºC. The formazan salts formed were then solubilised using DMSO. The results were quantified spectrophotometrically at 570 nm. This procedure was employed to establish the effects on total cell viability of treatments with Bio-LTT1$_{Ct}$ (cultures of either neurons or astrocytes) and NMDA (cultures of astrocytes). Only for the analysis of RIP contribution to excitotoxicity, we specifically calculated neuronal viability. As mentioned before, neuronal cultures contained a small percentage of glial cells, resistant to the excitotoxic damage [3, 4]. Therefore, we needed to calculate the contribution of glial viability to the total values. Thus, 24 h before the MTT assay, sister cultures were treated with 400 µM NMDA and 10 µM glycine, conditions that induced a complete neuronal death but no glial damage. The obtained absorbance value (glial viability) was then subtracted from total values to calculate the specific viability of neurons present in the cultures.

Subcellular fractionation

Cell cultures were washed twice at 4 °C with Tris Natrium-EDTA buffer (TNE, 150 mM NaCl, 50 mM Tris HCl pH 7.5, and 1 mM EDTA) and collected in the same buffer. A small aliquot of cell suspension was separated to prepare the total lysate (TL), while the remaining was used for cell fractionation to obtain the post-nuclear (Post-N) and nuclear (N) phases. The TL was prepared in NPBD buffer (10 mM NaCl, 10 mM Tris HCl pH 7.5, 2 mM $MgCl_2$, 0.5 % NP40, 0.1 % SDS, and 1 % sodium deoxycholate), containing protease and phosphatase inhibitors as before. After a 30 min incubation at 4 °C, the lysate was sonicated using a Bioruptor apparatus (Diagenode) and the cellular debris were eliminated by centrifugation at 3000 rpm for 10 min at 4 °C to obtain the final protein suspension. The Post-N phase was obtained by mild cell lysis with NPB buffer (10 mM NaCl, 10 mM Tris HCl pH 7.5, 2 mM $MgCl_2$, and 0.5 % NP-40), containing 2 mM DTT and protease and phosphatase inhibitors, conditions that maintained the nuclear integrity. After a 30 min incubation at 4 °C, the nuclei were separated from the Post-N fraction by centrifugation. Once the supernatant was collected (Post-N), the nuclei were washed twice with NPB buffer containing 2 mM DTT and the inhibitors, and then lysed with NPBD buffer, followed by sonication. After removal of nuclear debris by centrifugation, the N phase was collected. Compared to TL and Post-N, nuclear samples were more concentrated to facilitate TrkB-T1-ICD visualization. All fractions were analysed by BCA to determine protein concentration, fractioned by SDS-PAGE and analysed by immunoblot.

Immunoblot analysis

Cells were lysed in RIPA buffer (50 mM Tris-HCl pH 8.0, 150 mM NaCl, 1 % sodium deoxycholate, 1 % NP-40, 0.1 % SDS, and 1 mM DTT), supplemented with protease and phosphatase inhibitors (see Table S1), for 30 min at 4 ºC. Then, cell lysates were sonicated using a Bioruptor apparatus and

centrifugated at 4 ºC for 20 min at 11,000 rpm. The protein concentration in lysates was measured by using a BCA Protein Assay Kit (Thermo Fisher) followed by 5 min denaturalization in SDS-sample buffer at 95 ºC. Equal amounts of total lysates were resolved in Tris-Glycine SDS-PAGE and transferred to a nitrocellulose membrane (GE Healthcare) in 25 mM Tris HCl pH 8.3, 250 mM glycine and 10 % methanol, using an electric current of 400 mA for 70 min. After transfer, membranes were stained with a Ponceau S solution to check efficacy. Then, membranes were blocked for 30 min with a 5 % non-fat dry milk solution in Tris Buffered Saline-Tween (TBS-T, 20 mM Tris HCl pH 7.5, 137 mM NaCl, and 0.05 % Tween-20) and incubated overnight at 4 °C with primary antibodies (see Table S1). Next, membranes were washed with TBS-T and incubated with the appropriate anti-rabbit or anti-mouse peroxidase-conjugated secondary antibodies (Bethyl) for 1 h. Immunoreactivity was detected using Clarity Western ECL Blotting Substrate (BioRad) and band intensity was quantified by densitometric analysis (Photoshop, Adobe). Levels of GFAP were normalized using those of □-actin present in the same sample and expressed relative to the maximum values obtained in each experiment, arbitrarily given a 100 % value.

Cell transfection and gene reporter assays

Plasmids used contained minimal CREB or MEF2 response elements upstream firefly luciferase reporter genes (respectively, pCRE or pMEF2; see details in Table S1). Primary cultures, without AraC treatment, were transfected at 11 DIVs with 0.4 µg of those plasmids using Lipofectamine 2000. DNA-liposomes complexes were prepared according to the manufacturer's instructions in neurobasal medium (Thermo Fisher Scientific) with 1 mM Glutamax (Gibco, Thermo Fisher Scientific) and added to the cell cultures. After 2 h of transfection, this mix was replaced with previously collected conditioned medium and incubation proceeded to complete 24 h. Protein extracts were obtained using Passive Lysis Buffer (Promega, Cat# E1941) and luciferase activity was detected by using a luminometer (GloMax 96 microplate luminometer, Promega), samples being in REAP buffer (25 mM glycylglycine, 15 mM SO_4Mg, 4 mM EGTA, 15 mM potassium phosphate pH 7.8, 3.3 mM ATP, 1 mM DTT, and 75 µM luciferin).

RNA extraction and qPCR assay

Total RNA was extracted using QIAcube technology and treated with DNases before cDNA synthesis by the High-Capacity cDNA Reverse Transcription Kit (Applied Biosystems). A 7900HT Fast real-time PCR system (Applied Biosystems) was used for SYBR green gene expression assays (primers indicated in Table S1). PCR conditions were 10 min at 95 ºC, followed by 40 cycles of 15 s at 95 ºC and 60 s at 60 ºC. For each independent experiment, we made a specific standard curve for every gene and technical triplicates were prepared for every sample. Results were normalized to the levels of NSE or GAPDH genes as indicated.

Peptide visualization in primary neural cultures and immunocytochemistry

Primary cultures, seeded at half of the concentration previously indicated, were grown for 13 DIVs on coverslips previously treated overnight at 37 °C with poly-L-lysine and laminin as described before. In experiments designed for peptide visualization, cells were incubated with either Bio-TMyc or Bio-$LTT1_{Ct}$ (25 µM, 30 min), or left untreated, and then fixed for 30 min with 4 % paraformaldehyde in PBS. The fixed cells were washed several times with PBS before they were blocked and permeabilized for 4 h with 4 % goat serum and 0.5 % Triton X-100 prepared in PBS, respectively. Afterwards, coverslips were incubated overnight at 4 °C with the TrkB-T1 isoform-specific antibody diluted in the same blocking solution. After washing with PBS, the coverslips were incubated with the corresponding secondary antibody together with Fluorescein Avidin D (200 µg/ml) for 1 h at room temperature and then 10 min with DAPI to stain DNA. Finally, the samples were mounted in Prolong Diamond. Images were acquired using an inverted Zeiss LSM 710 laser confocal microscope (Jena, Germany) with 40x and 63x Plan-Apochromatic oil immersion objectives. Images corresponding to either single sections or maximum intensity projections were normalized for each colour separately and processed with ImageJ (NIH Image) and Fiji. For immunocytochemistry of cultures treated with NMDA, cells were fixed as described before and incubated with anti-TrkB-T1 antibodies alone (astrocytes) or together with anti-NeuN antibodies (neurons). Then, the procedure continued as indicated above but without incubation with Fluorescein Avidin D.

Peptide visualization in mice brain and immunohistochemistry

After intranasal (i.n.) peptide administration for 1 or 48 h, animals were deeply anesthetized and intracardially perfused with cold PBS and 4 % paraformaldehyde in PBS. Brains were postfixed in the same fixative at 4 °C for 24 h and cryoprotected in 30 % sucrose for 48 h at 4 °C. Floating coronal frozen sections (30 µm thick) obtained using a cryostat (Leica, Heidelberg, Germany) were incubated in blocking solution (10 % goat serum, 5 % BSA, and 0.5 % Triton X-100 in PBS) for 3 h at room temperature. When indicated, sections were incubated overnight with NeuN in 4 % goat serum right after blocking and, after washing, with either Alexa Fluor 488 or 647-conjugated antibodies together with Cy5-Streptavidin (Cy5-Strep, 5 µg/ml) or Fluorescein Avidin D (FluorAv, 200 µg/ml) and DAPI (5 µg/ml) for 1 h. Finally, sections were mounted and dried on slides, and cover slipped with Prolong Diamond. Image acquisition was performed using a Cell Observer (Zeiss), for wide-field peptide imaging in whole coronal sections labelled with Cy5-Streptavidin, or with an inverted laser confocal microscope with a 63x Plan-Apochromatic oil immersion objective for those labelled with FluorAv. Images presented were acquired and processed equally for each condition and corresponded to single sections. Background was subtracted using vehicle-injected animals.

For immunohistochemistry, brains were processed and cryoprotected as described before. Coronal frozen sections were incubated in flotation with the blocking solution for 1 h at room temperature,

followed by an overnight incubation with the indicated primary antibodies diluted in 4 % goat serum and 0.3 % Triton X-100 in PBS at 4 °C. After washing, slides were incubated 1 h with secondary antibodies (Table S1) and DAPI (5 µg/ml). Sections were then washed in PBS, mounted on slides, dried for 15 min at 37 ºC on a hot plate or air dried overnight, and then cover slipped with Prolong Diamond. Confocal images were acquired with 40x and 63x Plan-Apochromatic oil immersion objectives and processed as described above.

Images used for Iba1-based morphological analysis were obtained using an inverted Stellaris 8 Leica confocal microscope with a 40x objective. A tile of approximately 12 x 6 fields from 30 µm-thick brain coronal sections, corresponding to the cortical area, was acquired. The z-stacks covered the sample width, with 40 % overlap between stacks. AIVIA software version 15.0.0.42239 was used for 3D reconstruction, segmentation and morphological measurements. The segmentation code used for the analysis is available as part of this Supplementary Material file.

References

1. Tejeda GS, Esteban-Ortega GM, San Antonio E, Vidaurre OGG, Díaz-Guerra M. Prevention of excitotoxicity-induced processing of BDNF receptor TrkB-FL leads to stroke neuroprotection. EMBO Mol Med. 2019;11:e9950. https://doi.org/10.15252/emmm.201809950.

2. Watson BD, Dietrich WD, Busto R, Wachtel MS, Ginsberg MD. Induction of reproducible brain infarction by photochemically initiated thrombosis. Ann Neurol. 1985;17:497-504. https://doi.org/10.1002/ana.410170513.

3. Choi DW. Glutamate neurotoxicity in cortical cell culture is calcium dependent. Neurosci Lett. 1985;58:293-7. https://doi.org/10.1016/0304-3940(85)90069-2.

4. Choi DW, Maulucci-Gedde M, Kriegstein AR. Glutamate neurotoxicity in cortical cell culture. J Neurosci. 1987;7:357-68. https://doi.org/10.1523/JNEUROSCI.07-02-00357.1987.

Table S1. Key reagents and resources with indication of sources and identifiers.

**REAGENT or RESOURCE**	**SOURCE**	**IDENTIFIER**
Antibodies		
Mouse monoclonal anti-**β-Actin**	Sigma-Aldrich	Cat#A5441<br>RRID: AB_10920058
Rabbit polyclonal anti-**C3d**	Dako	Cat#A0063<br>RRID: AB_578478
Rabbit polyclonal anti-**Calnexin**	Enzo Life Science	Cat#ADI-SPA-860<br>RRID:AB_10616095
Mouse monoclonal anti-**CD68**	Abcam	Cat#ab955<br>RRID:AB_307338
Rabbit monoclonal anti-**CREB**	Cell Signaling	Cat#9197<br>RRID:AB_331277
Rabbit monoclonal ani-**GFAP**	Millipore	Cat#MAB360<br>RRID:AB_11212597
Mouse monoclonal anti-**GFAP**	Millipore	Cat#G6171<br>RRID:AB_1840893
Rabbit polyclonal anti-**Iba1**	Wako	Cat#019-19741<br>RRID: AB_839504
Rat monoclonal anti-**LAMP2**	Abcam	Cat#ab13524<br>RRID: AB_2134736
Mouse monoclonal anti-RBFOX3/**NeuN**	Novus	Cat#NBP1-92693SS;<br>RRID:AB_1103747
Rabbit polyclonal anti-**TrkB-FL (C-ter region)**	Abcam	Cat#ab18987<br>RRID: AB_444716
Rabbit polyclonal anti-**TrkB-T1** (isoform-specific C-ter)	Custom made	
Goat anti-mouse IgG Alexa Fluor 546	Molecular Probes	Cat#A-11030;<br>RRID:AB_144695
Goat anti-mouse IgG Alexa Fluor 647	Molecular Probes	Cat#A-21236<br>RRID:AB_2535805
Goat anti-rabbit IgG Alexa Fluor 488	Molecular Probes	Cat# A11034<br>RRID: AB_2576217
Goat anti-rabbit IgG Alexa Fluor 546	Molecular Probes	Cat# A11035<br>RRID: AB_143051
Donkey anti-rabbit IgG-heavy and light chain-HRP	Bethyl	Cat#A120-108P;<br>RRID:AB_10892625
Donkey anti-mouse IgG-heavy and light chain-HRP	Bethyl	Cat#A90-137P;<br>RRID:AB_1211460
Chemicals		
**APMA** (used at 100 µM)	Calbiochem	Cat#164610<br>CAS: 6283-24-5
**Ara C** (used at 10 µM)	Sigma-Aldrich	Cat#C1768;<br>CAS: 147-94-4
**B27** serum free supplement	Thermo Fisher	Cat#17504044
**Cy5-Streptavidin**	Palex Medical SA	Cat#SA-1500-1
**DAPI** (used at 0.5 or 5 µg/ml)	Molecular Probes	Cat#D1306
**EGF** (Human epidermal growth factor recombinant prot**.)**	Thermo Fisher	Cat# PHG0311
b**FGF** (Human basic fibroblast growth factor recomb. prot.)	Thermo Fisher	Cat#13256-029
**Fluorescein Avidin** D	Vector Laboratories	Cat#A 2001
**Glycine** (used at 10 µM)	Bio-Rad	Cat#161-0718

**Laminin** (used at 4 µg/ml)	Sigma-Aldrich	Cat#L2020; CAS: 114956-81-9
**MTT** (used at 0.5 mg/ml)	Sigma-Aldrich	Cat#M5655; CAS: 298-93-1
**NMDA** (used at 100 µM)	Tocris	Cat#0114; CAS: 6384-92-5
**PhosSTOP** phosphatases inhibitor cocktail tablets	Roche	Cat#04 906 837 001
**Poly-L-Lysine** (used at 100 µg/ml)	Sigma-Aldrich	Cat#P1524; CAS: 25988-63-0
**Prolong** Diamond antifade reagent	Molecular Probes	Cat#P36970
Complete **protease inhibitor** cocktail tablets	Roche	Cat#11 697 498 001
**TAPI-2** (used at 10 µM)	Peptides International	Cat#INH-3852-PI CAS: 187034-31-7
Peptides		
**Bio-LTT1$_{Ct}$** (Biotin-Ahx-*YGRKKRRQRRR*KLARHSKFGMKGFVLFHKIPLDG-NH_2) M_W: 4507 Da	GenScript	N/A
**Bio-TMyc** (Biotin-Ahx-*YGRKKRRQRRR*AEEQKLISEEDLLR-NH_2) M_W: 3553 Da	GenScript	N/A
Primer sequences for real-time PCR		
GluN1-F (all isoforms): TCCACCAAGAGCCCTTCGTG	Sigma-Aldrich	N/A
GluN1-R (all isoforms): GCCCCGTACAGATCACCTTC	Sigma-Aldrich	N/A
GluN2A-F: ACGACTGGGACTACAGCCTG	Sigma-Aldrich	N/A
GluN2A-R: CTTCTCTGCCTGCCCATAGC	Sigma-Aldrich	N/A
TrkB-FL-F (isoform specific): TATCTTCACCCACCTCAAAC	Sigma-Aldrich	N/A
TrkB-FL-R (isoform specific): GAGAGACTTGACCTGAGCAC	Sigma-Aldrich	N/A
TrkB-T1-F (isoform specific): GGGGCTGTGCTGCTTGGT	Sigma-Aldrich	N/A
TrkB-T1-R (isoform specific): GCTGCGGACATCTTTGGAGA	Sigma-Aldrich	N/A
BDNF-F (all isoforms): TGGCTGACACTTTTGAGCAC	Sigma-Aldrich	N/A
BDNF-R (all isoforms): GTTTGCGGCATCCAGGTAAT	Sigma-Aldrich	N/A
GAPDH-F: TTGCCATCAACGACCCCTTC	Sigma-Aldrich	N/A
GAPDH-R: GCCTTGACTGTGCCGTTGAA	Sigma-Aldrich	N/A
NSE-F: ACAGAATGGGGCTGTGTACC	Sigma-Aldrich	N/A
NSE-R: TGGCAACTGTGGGACATGGC	Sigma-Aldrich	N/A
Critical Commercial Assays		
BCA Protein Assay Kit	Thermo Fisher	Cat# 23225
Clarity Western ECL Blotting Substrate	BioRad	Cat# 1705060
Lipofectamine 2000	Life Technologies	Cat#11668019
Experimental Models: Organisms/Strains		
Balb/c inbred mice (Balb/cAnNRj)	Janvier Labs SAS	N/A
Wistar Rat embryos (E18)	In site facility	N/A
Recombinant DNA		
**pCRE** (25-mer oligonucleotide with the sequence of two TrkB CREs subcloned into pTK-Luc)	(Deogracias et al., 2004)	N/A
**pMEF2** (pRSRF; -307 to -242 of *Nur77* promoter with two MEF2 sites subcloned into pGL2-basic)	(Woronicz et al., 1995)	N/A

**pMEF2mut** (two inactivating point mutations in each MEF2 site of pMEF2)	(Woronicz et al., 1995)	N/A
Software		
Adobe InDesign		RRID:SCR_021799
Adobe Photoshop		RRID:SCR_014199
AIVIA	version: 15.0.0.42239	
GraphPad Prism		RRID:SCR_002798
ImageJ	imagej.net/	RRID:SCR_003070
ProtNLS https://www.novoprolabs.com/tools/nls-signal-prediction		
R Project for Statistical Computing	www.r-project.org	RRID:SCR_001905
Other		
Tissue-Tek O.C.T Compound	Sakura	Cat#4583
Protran Western blotting nitrocellulose membrane	GE Healthcare	Cat#GE10600002; CAS: 9004-70-0

Deogracias R, Espliguero G, Iglesias T, Rodriguez-Pena A. Expression of the neurotrophin receptor trkB is regulated by the cAMP/CREB pathway in neurons. Mol Cell Neurosci. 2004;26:470-80. https://doi.org/10.1016/j.mcn.2004.03.007

Woronicz JD, Lina A, Calnan BJ, Szychowski S, Cheng L, Winoto A. Regulation of the Nur77 orphan steroid receptor in activation-induced apoptosis. Mol Cell Biol. 1995;15:6364-76. https://doi.org/10.1128/MCB.15.11.6364

Supplementary Figures

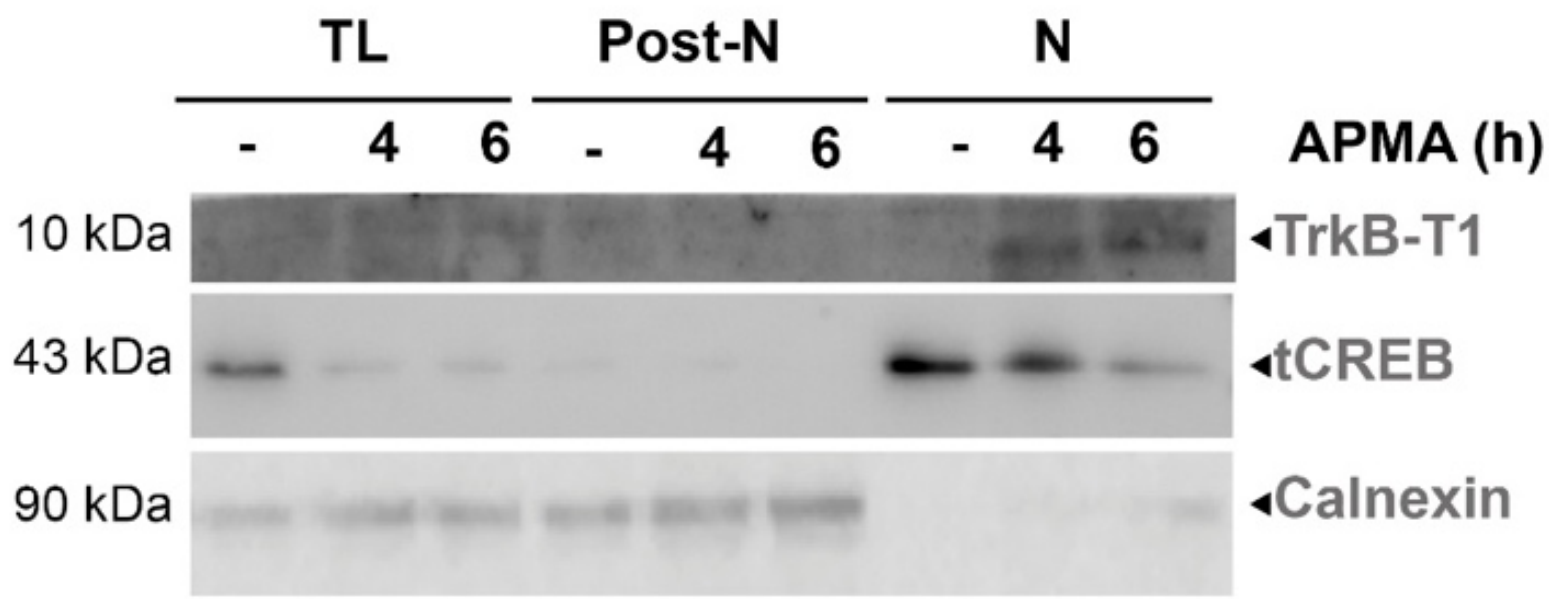

Fig. S1 Subcellular fractionation of cultured astrocytes treated with APMA. Cultures were incubated for 4 or 6 h with APMA (100 µM). TrkB-T1-ICD was detected by the TrkB-T1 isoform-specific antibody. Correct cell fractionation of total lysates (TL) was checked using calnexin as a Post-nuclear (Post-N) marker and tCREB as a nuclear (N) marker.

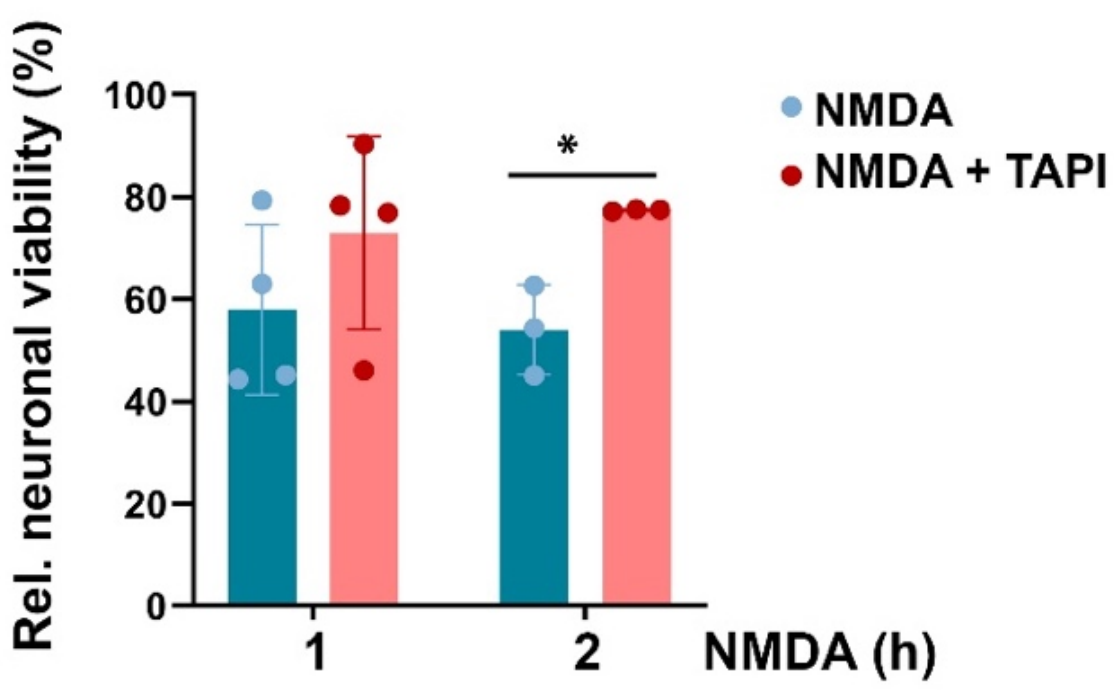

Fig. S2 Excitotoxicity-induced RIP contributes to neuronal death of primary cultures of cortical neurons. Neuronal viability was measured in cultures treated with NMDA (100 µM) for 1 and 2 h, with or without a 30 min pretreatment with a broad-spectrum metalloprotease inhibitor (TAPI-2, 10 µM). Individual data of neuronal viability relative to that of untreated cells are presented as the mean ± SEM (n = 3-4). Technical replicates were included in each independent experiment (N = 3). Data were statistically analysed using a paired Student's t-test.

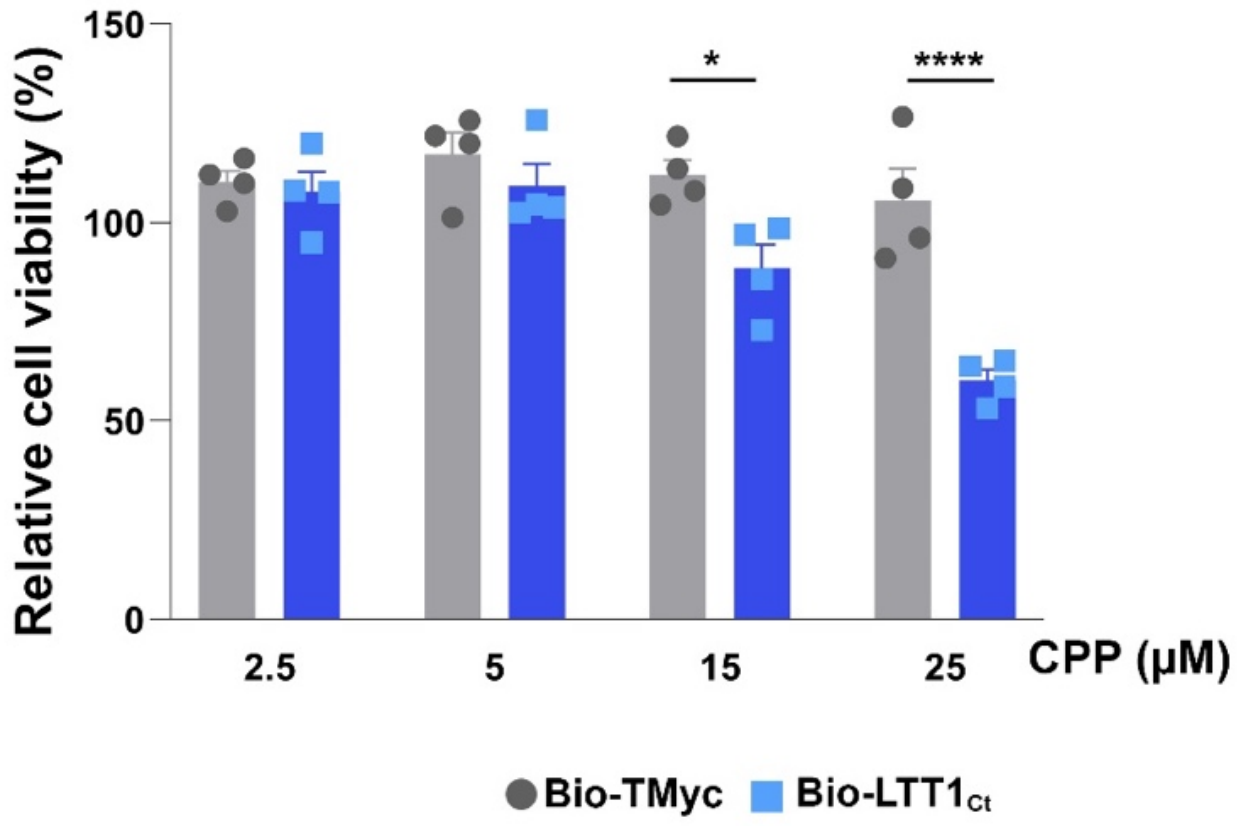

Fig. S3 Bio-LTT1Ct toxicity is dose-dependent. Cell viability was measured in primary neurons treated with 2.5, 5, 15 or 25 µM of Bio-LTT1Ct or Bio-TMyc for 4 h. Viability values are expressed relative to the viability obtained in cells treated with 25 µM Bio-TMyc (considered as 100 %). Individual values are presented and data are expressed as the mean ± SEM (*n* = 4). Technical replicates were included in each independent experiment (*N* = 3). Statistical analysis was performed by two-way ANOVA test followed by *post hoc* Sídák's test.

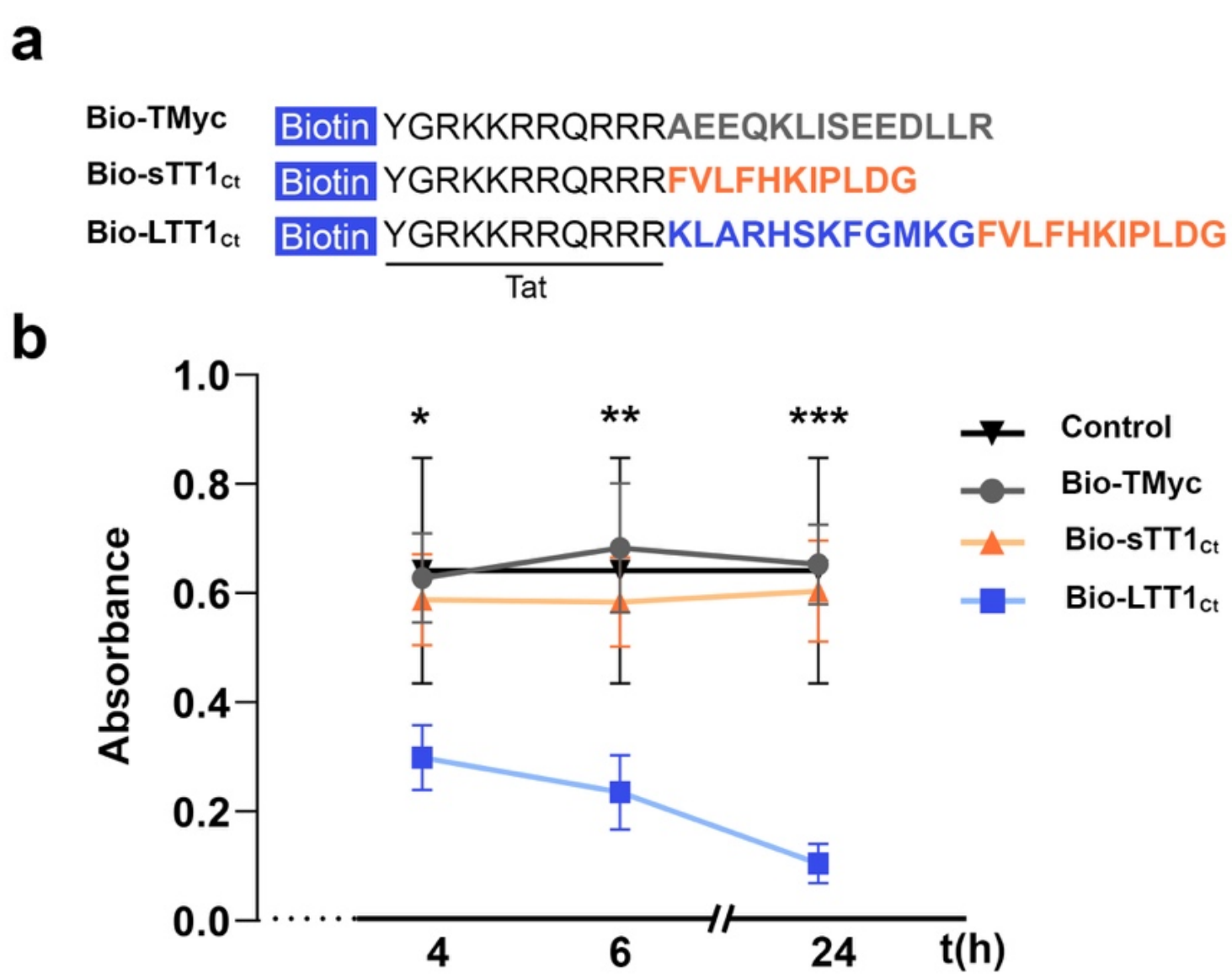

Fig. S4 Bio-LTT1Ct toxicity is specific to TrkB-T1 454-465 sequence. **a** Sequence comparison of TrkB-T1-derived peptides Bio-LTT1Ct and Bio-sTT1Ct. They share a biotin group at the N-terminal, followed by the indicated Tat sequence and isoform-specific amino acids 466 to 471 (orange). Differently from Bio-sTT1Ct, Bio-LTT1Ct also includes TrkB-T1 intracellular amino acids 454-465 (blue). The sequence of the control peptide Bio-TMyc is also indicated. **b** Comparison of Bio-LTT1Ct and Bio-sTT1Ct (25 µM) effects on cell viability. Before the MTT assay, primary neurons were incubated with Bio-TMyc or the TrkB-T1-derived peptides for 4, 6 or 24 h or, alternatively, left untreated. The absorbance at 570 nm of the solubilised formazan salts formed after MTT reduction is presented and data are expressed as the mean ± SEM (*n* = 3-4). Technical replicates were included in each independent experiment (*N* = 3). Statistical analysis was performed by two-way ANOVA test followed by *post hoc* Tukey's test (* for Bio-LTT1Ct *vs* Bio-sTT1Ct).

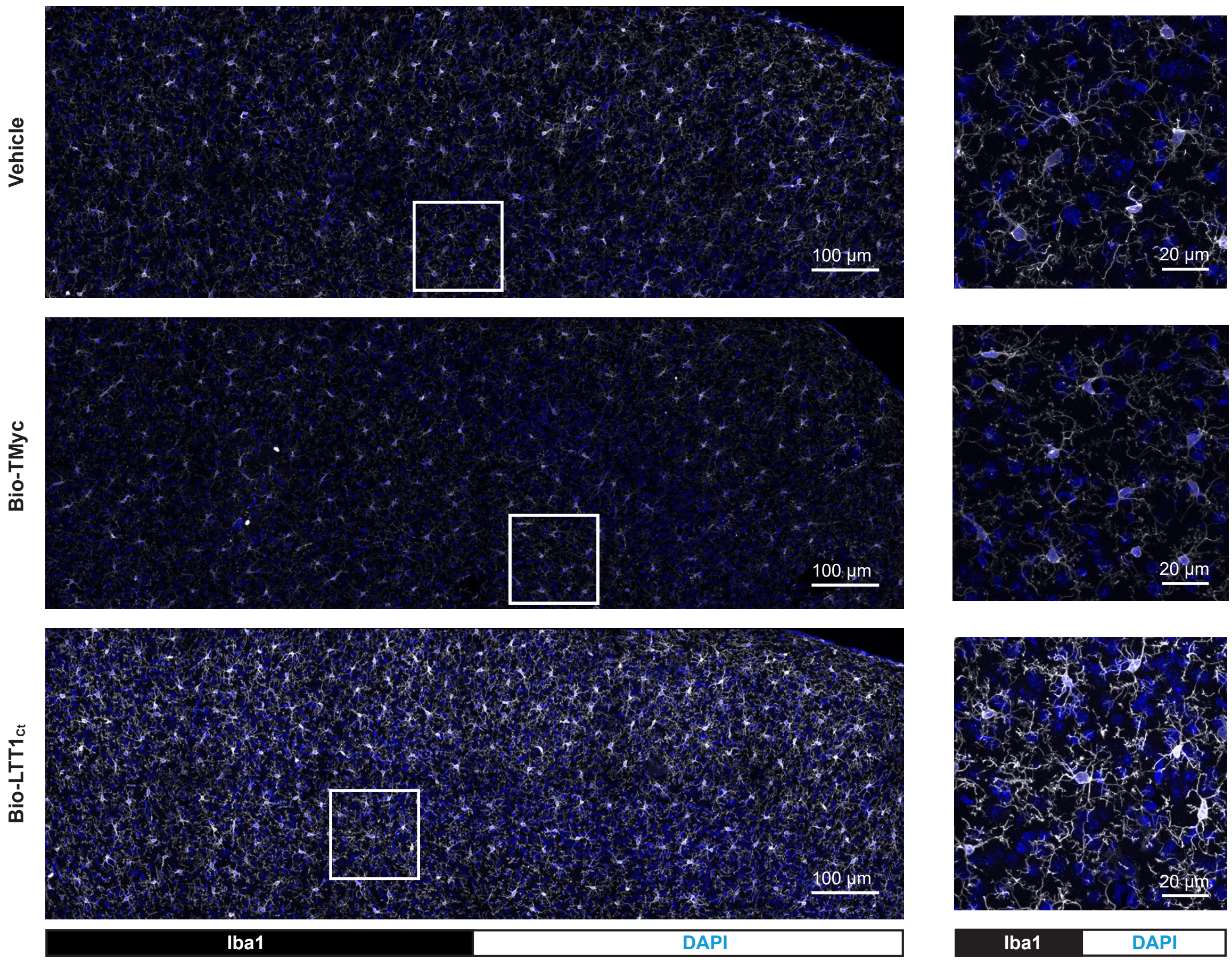

Fig. S5 Microglia morphological analysis. Representative Iba1 and DAPI staining of complete tiles from the cortical area of animals treated with vehicle, Bio-TMyc or Bio-LTT1$_{Ct}$. The fields selected for display in Fig. 7A are indicated with a white square and enlarged next to its corresponding tile. Scale bars are indicated in each case.

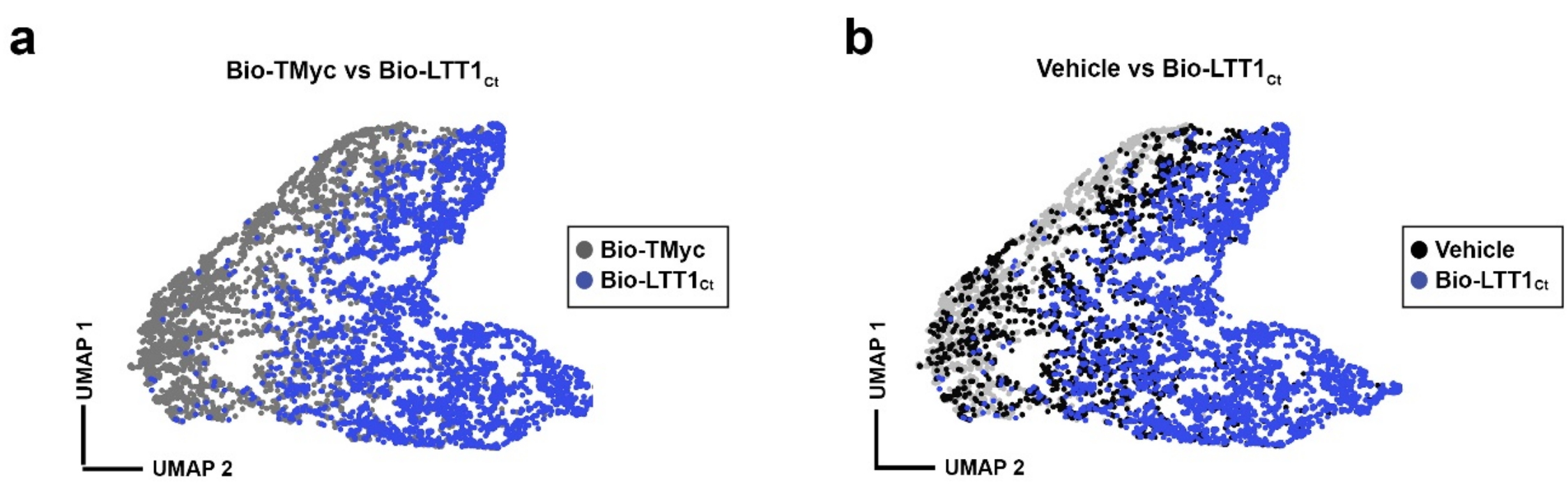

Fig. S6 UMAP plot represents microglial cells from all mice and groups of treatment. Cells are presented using UMAP1 and UMAP2 dimensions. **a** and **b** panels highlight cells from the Bio-LTT1$_{Ct}$ treated animals (blue) and those from animals treated with Bio-TMyc (grey) or vehicle (black), respectively.

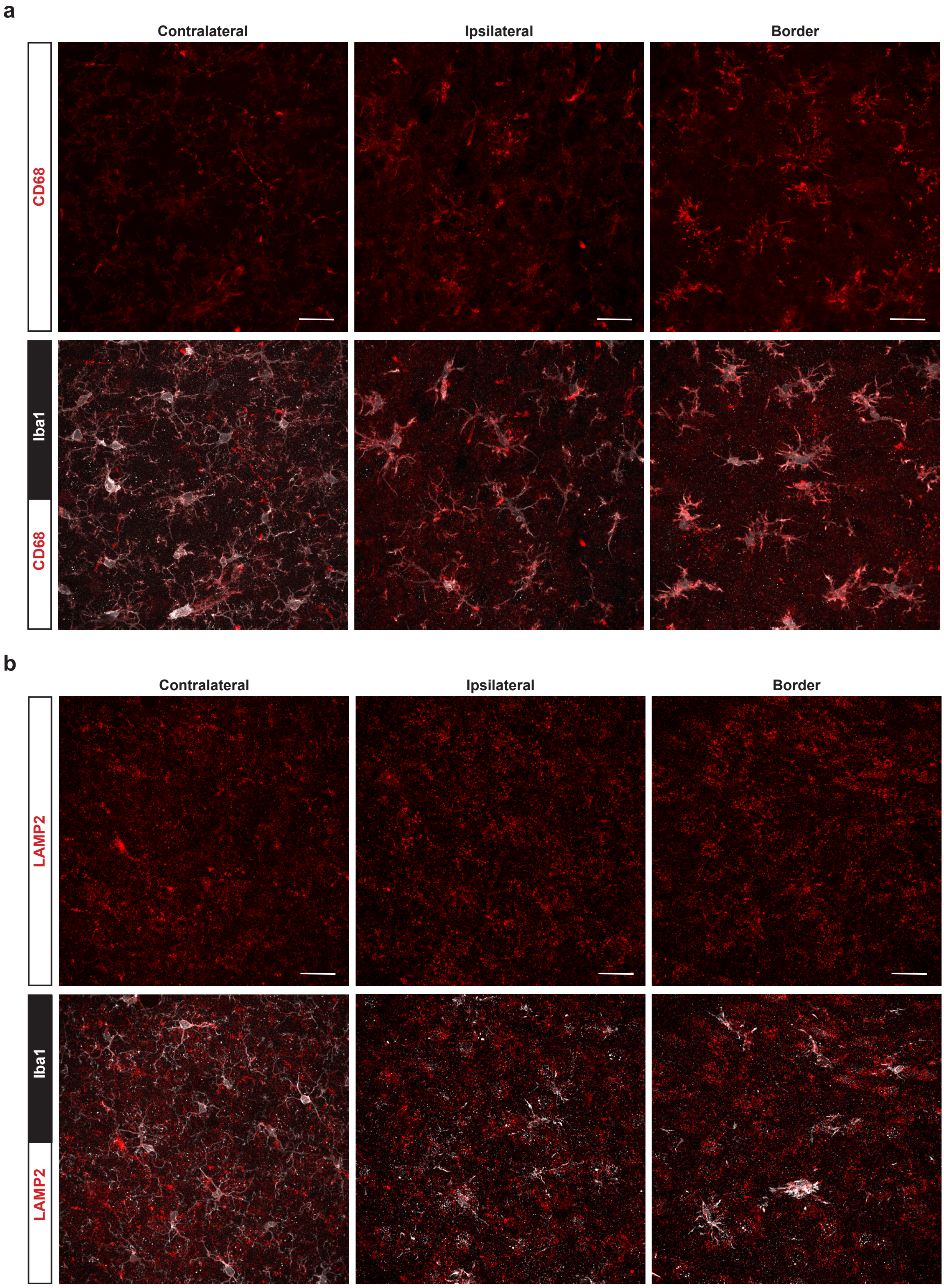

Fig. S7 Microglia proinflammatory profile in an experimental *in vivo* model of stroke. Immunofluorescence of Iba1 (white) and CD68 (red) (**a**) or LAMP2 (red) (**b**), 5 h post-ischaemia. Representative confocal images from the cortical areas corresponding to the infarct border, ipsilateral and contralateral regions of injured male mice are presented. Scale bars represent 20 µm, n=3 per group.

Segmentation code used for analysis

15.0.0.42239
1

6ff29774-9810-4fb7-abe2-e67642c5a517
1

3D Object Analysis - Meshes
ddc70a23-5121-4110-bcc6-0599e3b1d67e

3D Object Analysis - Meshes
Detection
7ab108c8-ac0f-4601-9dc0-108707305e37

3D Object Analysis - Meshes
Partition
4f471a17-2942-4d0f-b919-0edf74e11c5d

Max Object Radius
27e90136-bdb0-4c51-b37f-c6b75ec5a57a
200

Min Object Radius
6883d75d-bdb7-459d-8213-3e6a1b8c1e65
5

Mesh Smoothing Factor
ffd11422-6b54-4837-93a9-e9e454358404
0

Skip Smooth Image
Enhance Cells
8e49dc27-1f25-4f3a-aa5c-9ac7384baf78

Image Smoothing Filter Size
baf5f5e3-23df-43f1-9fc0-82c087818e4b
0

Morphological Smoothing
Smooth Cells
52a8b407-b8d2-4cfe-9683-b24948746904

Image Smoothing Filter Size
8bc07246-a5c6-4f43-9f10-1fd0ac167bb3
1

Average Filter Smoothing

Smooth Cells
52a8b407-b8d2-4cfe-9683-b24948746904

Image Smoothing Filter Size
8bc07246-a5c6-4f43-9f10-1fd0ac167bb3
9

Gaussian Filter Smoothing
Smooth Cells
52a8b407-b8d2-4cfe-9683-b24948746904

Image Smoothing Filter Size
8bc07246-a5c6-4f43-9f10-1fd0ac167bb3
9

Median Filter Smoothing
Smooth Cells
52a8b407-b8d2-4cfe-9683-b24948746904

Image Smoothing Filter Size
8bc07246-a5c6-4f43-9f10-1fd0ac167bb3
9

Skip Remove Background
Object Detection
a5d14608-cc7d-436c-97c3-cc511ba2a08d

Min Edge Intensity
36d6211a-0944-4bc3-9ea6-e9e2f18236ae
0.00784313725490196

Fill Holes Size
333ec50c-2d60-4ee4-912d-f5864b73c9a3
0

Remove Background
Object Detection
a5d14608-cc7d-436c-97c3-cc511ba2a08d

Average Object Radius
ea132080-a499-40a1-9035-93f7ce144cdf
20

Min Edge Intensity
f1c400e1-d376-4fb4-ad27-27608242db0d
0.12

Fill Holes Size
1fea40e0-5167-4a03-b6ca-fd12703b5d4e

0

Apply Partition
Partition
aa76e840-fe29-4414-93c3-ecd47d3b9533

Min Edge to Center Distance
a62966fc-39de-43fa-ab97-7362df00cb07
20

Skip Partition
Partition
a949756a-c62e-4b2d-8c12-33620011ac7d

Min Edge to Center Distance
f7b6e05a-d0ad-441e-9bd6-7673bf246de5
20

Smoothing_WholeCell_switchable
Morphological Smoothing

ImageEnhancementOptions
Skip Remove Background

ObjectPartitionOptions
Apply Partition

Cross Sections
454067e5-9dce-4387-80eb-fb1ad7be2e01
true
